# Alterations in ascending aortic hemodynamics and aortic length correlate with sex-specific thoracic aortic aneurysm dilation and lifespan in a mouse model of severe Marfan syndrome

**DOI:** 10.1016/j.compbiomed.2026.111594

**Published:** 2026-03-02

**Authors:** Krashn Kr Dwivedi, Yufan Wu, Marisa S. Bazzi, Hadi Wiputra, Jacob Rother, James D. Quirk, Victor H. Barocas, Jessica E. Wagenseil

**Affiliations:** a Department of Mechanical Engineering and Materials Science, Washington University, Saint Louis, MO, USA; b Department of Pediatrics, Stanford University, Palo Alto, CA, USA; c Department of Biomedical Engineering, University of Minnesota, Minneapolis, MN, USA; d Mallinckrodt Institute of Radiology, Washington University School of Medicine, Saint Louis, MO, USA

**Keywords:** Aortic aneurysm, Fluid-structure interaction, Extracellular matrix, Biomechanics, Hemodynamics

## Abstract

Thoracic aortic aneurysm (TAA) is a dilation of the aorta that may eventually dissect and/or rupture. It is associated with genetic disorders such as Marfan syndrome (MFS) and is a life-threatening cardiovascular condition if left untreated. Current clinical guidelines for TAA management are primarily based on maximum diameter thresholds that are often inadequate, particularly in MFS patients. Moreover, the diameter thresholds are not sex-specific, despite growing evidence that TAA outcomes in MFS are influenced by sex. The aim of this study was to identify non-invasive biomarkers for better management of TAA using male and female mice that are a genetic model of severe MFS and their littermate controls. Fluid–structure interaction (FSI) simulations were performed to assess aortic geometry, hemodynamics, and wall mechanical stresses during TAA progression (as measured by aortic dilation) and outcomes (as measured by mouse lifespan). Oscillatory shear index (OSI) correlated significantly with TAA progression in males, but not females, while time averaged wall shear stress (TAWSS) correlated significantly with TAA progression in females, but not males. Endothelial cell activation potential (ECAP), a metric that combines OSI and TAWSS, was significantly correlated with TAA progression in both sexes and had the strongest correlation with lifespan of all hemodynamic metrics. The geometric metric of aortic elongation ratio (AER) (i.e. length) also had strong correlations with TAA progression and lifespan in male and female mice. This study demonstrates that hemodynamic and geometric metrics hold promise as noninvasive biomarkers for personalized management of TAA in MFS.

## 1. Introduction

Thoracic aortic aneurysm (TAA) is a cardiovascular condition that affects approximately 5.3 out of every 100,000 people annually, with a mortality rate exceeding 90% if left untreated [1]. TAA is associated with genetic disorders such as Marfan syndrome (MFS). MFS is a multisystem connective tissue disorder caused by mutations in the fibrillin-1 (FBN1) gene [2]. The aorta in MFS is characterized by extracellular matrix (ECM) remodeling, altered smooth muscle cell phenotype, and dysregulated transforming growth factor-beta signaling [3]. These changes weaken the aortic wall, promoting the development and progression of TAA, which can ultimately dissect and/or rupture (i. e. fail) [4]. Surgical intervention to prevent TAA failure in MFS is recommended when the aortic diameter reaches 5 cm in the absence of other risk factors, however approximately 40% of patients experience aortic events such as dissection or rupture at diameters below this threshold [5]. Additionally, this criterion does not offer accurate guidance for sex-specific risk assessment and management of TAA [6]. Aortic diameter alone does not account for critical factors such as local hemodynamics, wall stress distribution, mechanical properties, and ECM remodeling that play a role in wall failure. This highlights the need for additional criteria to guide interventional decisions in TAA.

Ours [7–11] and others [12–14] previous work using genetic mouse models of MFS demonstrated that geometrical, microstructural and biomechanical metrics correlate with TAA progression, indicating their potential as biomarkers for TAA management. Several studies have shown that changes in biofluidic metrics such as luminal pressure, wall shear stress, and intramural stress correlate with TAA outcomes and these parameters can be determined non-invasively using imaging and computational modeling, making them promising biomarkers for TAA management in mice [7,15] and humans [16,17]. Despite documented sexual dimorphism in TAA progression and outcomes, the sex-specific impact of changes in biofluidic metrics and their connection to TAA remains poorly understood. Elucidating these differences is critical for the identification of patient-specific biomarkers to improve TAA management in MFS.

In this study, we investigated age- and sex-specific effects of MFS on aortic geometry and biofluidic metrics in mice, with the goal of identifying potential biomarkers for the management of TAA. We used $Fbn1^{mgR/mgR}$ and wild type (WT) male and female mice. $Fbn1^{mgR/mgR}$ is a fibrillin-1 deficient mouse model which mimics clinical features of MFS, shows early TAA development, and reduced lifespan [18]. Longitudinal magnetic resonance (MR) images of the entire aorta and the blood flow rate at the inlet of the ascending aorta were collected at ages 1, 2, 3 and 4 months. The MR data and ex vivo aortic wall mechanics [11] were used to build mouse-specific fluid structure interaction (FSI) models to determine the biofluidic metrics [7]. ECM remodeling was assessed using multiphoton microscopy [8,10], as hemodynamic stresses may play a role in ECM degradation and contribute to TAA progression and adverse outcomes [19,20]. Correlations with TAA progression and outcomes, as measured by aortic dilation and mouse lifespan, respectively, were used to identify possible biomarkers. Our study revealed that hemodynamic and geometric metrics correlate with sex-specific TAA progression and outcomes in mice and suggests further research to determine if they may be useful biomarkers for clinical TAA management.

## 2. Materials and methods

**Fig. 1.**
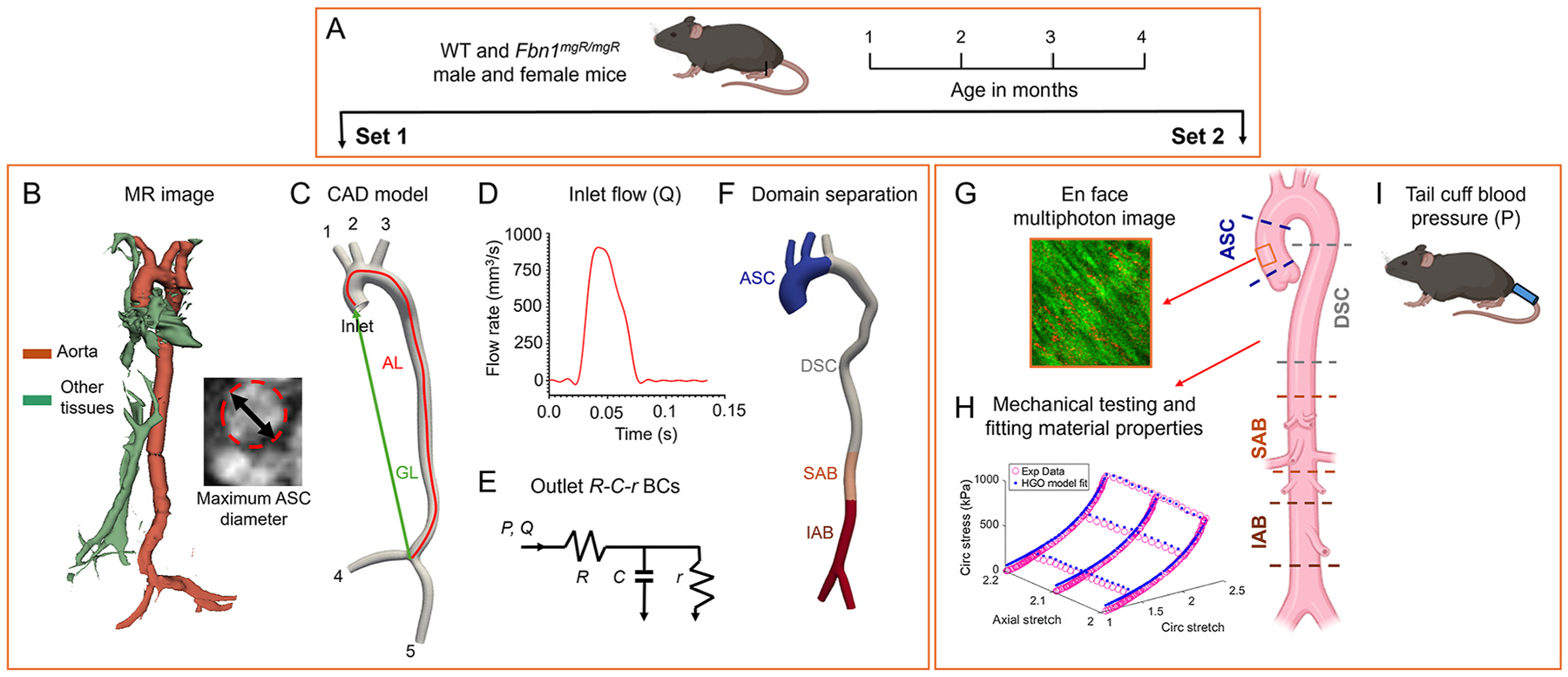
Pictorial presentation of the study design. Two sets of WT and $Fbn1^{\text{mgR}/\text{mgR}}$ (MU) male and female mice were used at ages 1–4 months (A). For Set 1 mice, MR images (B) were recorded in the same mice at each age. A CAD model was generated from the segmented data (C) and the maximum diameter and aortic elongation ratio (AER) from the actual length (AL) divided by the geometric length (GL) were measured from the centerline. The inlet flow at the aortic root was measured using MR imaging (D) and the R-C-r values for the Windkessel model at the outlets (1–5) were optimized using flow and pressure data (E). The aorta was divided into four domains along its length: ascending (ASC), descending (DSC), superior abdominal (SAB), inferior abdominal (IAB) (F) for assignment of region-specific material properties. For Set 2 mice, ex vivo data were gathered from different mice at each age. En face multiphoton images were used to measure elastic fiber degradation (G) and biaxial mechanical testing was used to determine the wall mechanical properties for each aortic region (H). Blood pressure was measured using the tail cuff method (I) before sacrifice. Data from Set 2 mice are included in our previously published papers [9–11].

We employed experimental and computational approaches to assess changes in aortic geometry, flow rate, blood pressure, ex vivo wall mechanics, ECM remodeling, and fluid-solid mechanics during TAA progression and failure. Fig. 1 is an overview of the study design. Two different sets of mice were used to collect longitudinal data. For Set 1 mice, we collected in vivo data from the same mice at multiple time-points. We performed in vivo longitudinal MR imaging of the aorta (Fig. 1B), segmented the lumen into a 3D computer-aided design (CAD) model (Fig. 1C), applied inlet (Fig. 1D) and outlet pressure and flow boundary conditions, tuned Windkessel R-C-r parameters to incorporate the downstream flow resistance (Fig. 1E), separated the 3D model into four domains to enable region-specific material properties (Fig. 1F), and then performed FSI simulations using SimVascular [21]. For Set 2 mice, we collected ex vivo data from different mice at each timepoint. En-face multiphoton images were recorded to characterize ECM organization (Fig. 1G) and mechanical testing was performed to determine region-specific material properties (Fig. 1H) of the aortic wall. Before sacrifice, the tail cuff method was used to measure blood pressure (Fig. 1I). Additional methodological details are provided below.

### 2.1. Mice

All protocols were approved by the Washington University Institutional Animal Care and Use Committee (IACUC) and conform to the current NIH guidelines. We used a severe mouse model of MFS ($Fbn1^{\text{mgR}/\text{mgR}}=\text{MU}$) [18]) and their wild-type (WT) littermates. MU and WT mice were obtained from breeding pairs of $Fbn1^{mgR/+}$ male and female mice. Mice were backcrossed >10 generations onto a C57BL6J background and refreshed by backcrossing every three generations. Data were collected from MU and WT male and female mice at the ages of 1, 2, 3, and 4 months of age (mo) (Fig. 1A). In vivo longitudinal data on aortic geometry and flow rate from MR imaging were obtained from the same mice at each timepoint for Set 1 (Fig. 1B–F) starting at 1 mo and imaging every month until 4 mo or death, whichever came first. In Set 1, we included a total of 17 mice (3 WT males, 3 WT females, 6 MU males and 5 MU females).

Due to the severity of the disease phenotype, the expected lifespan of this MFS model is approximately 2–4 mo for MU males and 3–8 mo for MU females [9]. As a result, maintaining a constant number of MU mice across all time points in this longitudinal study was challenging. In the present cohort, 5 of 11 MU mice (3 males and 2 females) in Set 1 died before reaching 4 mo. The death of the mice reflects the aggressive nature of this disease model and was accounted for to the best of our ability in the statistical analyses.

For computational simulation, the measurements of blood pressure, ECM remodeling, and ex vivo wall mechanics were collected for different sets of mice (Set 2) at each timepoint (Fig. 1G–I). Set 1 mice that survived to 4 mo were sacrificed for Set 2 studies, hence there is some overlap in the mice used in each set for the 4-mo timepoint. Data from Set 2 mice were included in our previously published papers [9–11].

### 2.2. Magnetic resonance (MR) imaging

MR images were acquired using a 40 mm ID quadrature RF coil on a 9.4 T MRI (Bruker BioSpec 94/20 USR, Ettlingen, Germany) running ParaVision 360 version 3.5. During MR imaging, the mice were anesthetized with 1 – 2% isoflurane and imaged in the supine position. Respiratory rate, ECG, and temperature were monitored (SA Instruments, Stony Brook, NY), and the temperature was maintained with warm air, gated off the rectal temperature.

Axial time-of-flight angiography images for in vivo aortic geometry (Fig. 1B) were acquired with prospective cardiac and respiratory gating from the aortic arch to the kidneys with the following parameters: TR/TE=15/2.7ms, flip angle = 90°, 2 averages, matrix = 214×107, field of view = 32 × 16 mm^2^, 230–260 overlapping slices of 0.3 mm thickness for a nominal resolution of 0.15×0.15×0.15 mm^3^.

Velocity maps in the ascending aorta for inlet blood flow (Fig. 1D) were acquired with prospective cardiac and respiratory gating using the following parameters: TR/TE=7.33/2.54ms, flip angle = 20°, 16 averages, matrix = 192×96, field of view = 32 × 16 mm^2^, single 0.8 mm slice, 15 frames/cardiac cycle. The maximum velocity was set to 150 cm/s.

### 2.3. 3D aorta model construction

FSI simulations require two distinct domains: a fluid domain, representing the aortic lumen, and a solid domain, corresponding to the aortic wall. The aorta was segmented from MR images (Fig. 1B), and an initial 3D model of the lumen was generated using SimVascular [21]. This lumen model included the brachiocephalic trunk, the left common carotid artery, the left subclavian artery, and the iliac arteries (Fig. 1C). To ensure geometric fidelity, the model was smoothed at vascular bifurcations within SimVascular, with additional refinements performed using Meshmixer (Autodesk Inc.). The aortic wall (solid domain) was generated by extruding the lumen wall outward by a radial distance equal to the diastolic wall thickness in Meshmixer (Autodesk Inc.). Due to the limited spatial resolution of MR imaging and the very thin wall of the mouse aorta, direct measurement of the aortic wall thickness was not feasible. Consequently, sex-, genotype-, and age-specific diastolic wall thickness values of the ascending aorta (ASC) were derived from ex vivo measurements of Set 2 mice [9]. In brief, we measured the loaded outer dimensions of the ASC at different pressures and axial stretches from biaxial mechanical tests. We measured the unloaded dimensions of the ASC from cut rings. The wall thickness at the diastolic pressure and in vivo axial stretch ratio was calculated assuming incompressibility [10]. The aortic wall thickness was kept uniform over the entire length of the aorta. These two domains were used to generate the mesh for FSI simulations.

The maximum diastolic diameter of the ASC and the aortic elongation ratio (AER) were measured from the 3D model of the aortic lumen. The centerline of the lumen geometry was extracted using SimVascular and further processed with a custom-written Python script. The diastolic aortic inner diameter from the aortic root to the brachiocephalic artery was measured from each MR image slice using a best-fit circle perpendicular to the center line to determine the maximum ASC diameter as a measure of TAA progression (Supplementary Fig. S1). AER was defined as the ratio between the actual (centerline) length (AL) and the geometric (straight line) length (GL) of the extracted aortic centerline from the aortic root to the iliac bifurcation (Fig. 1C).

### 2.4. Microstructural characterization of the aortic wall

Prior studies showed that the ASC region is particularly susceptible to TAA formation and marked ECM degradation [9,10,13,14] that may be linked to hemodynamic stress [19,20]. As a result, our microstructural analyses focused exclusively on ECM remodeling in the ASC and particularly on elastic fiber degradation or the loss of elastic fibers and on medial collagen fiber deposition. Data on elastic fiber porosity at 1, 2, 3, and 4 months for 3 mice/group from Set 2 were obtained by en face multiphoton imaging (Fig. 1G), following the protocol detailed in our previous study [10]. The medial collagen fiber content was also obtained from en face multiphoton imaging and was previously published [9].

### 2.5. Fluid-structure interaction (FSI) simulations

#### 2.5.1. Mesh generation

Both the fluid (lumen) and solid (wall) domains were meshed using tetrahedral elements in SimVascular [21], employing the TetGen meshing tool. To ensure proper enforcement of kinematic and dynamic boundary conditions at the fluid-structure interface, the mesh nodes on the fluid and solid domains were kept matching at the interface. The final mesh element size for the simulation was selected based on a mesh convergence test, ensuring that any further refinement in element number resulted in less than a 5% change in systolic wall shear stress. Furthermore, the solid domain was partitioned into four subdomains: ASC, descending (DSC), superior abdominal (SAB), and inferior abdominal (IAB) based on their anatomical locations (Fig. 1F) [10], to enable the assignment of region-specific material properties to the aortic wall.

#### 2.5.2. Blood properties

The rheological behavior of blood was described as non-Newtonian using the shear thinning Carreau-Yasuda model (Eq. (1)) [7],

Eq. 1 $$\eta =\eta_{\infty }+\left(\eta_{0}-\eta_{\infty }\right){\left(1-(\mu \dot{\gamma }{)}^{a}\right)}^{\frac{n-1}{a}},$$

where η is the apparent viscosity which depends on shear rate $\dot{\gamma }$ and $\eta_{\infty }$ and $\eta_{0}$ are the blood viscosities corresponding to infinite and zero shear rates, respectively. The parameters μ,a and n describe the power-law region between the plateaus at infinite and zero shear rates. The values ($\eta_{\infty }=2cP,\eta_{0}=11cP,\lambda =1.5,a=0.2$, and n=0.71) were obtained from our previous analysis of healthy human blood [22].

#### 2.5.3. Aortic wall mechanical properties

The mechanical properties of the aortic wall vary along its length, which is physiologically essential for regulating pulsatile blood flow and maintaining central blood pressure. Region-specific properties influence pulse wave propagation [23], which can in turn affect flow patterns and hemodynamics. To capture this behavior in the simulations, region-specific mechanical responses of the aortic wall corresponding to the ASC, DSC, SAB, and IAB were modeled as nearly incompressible (Poisson ratio ν=0.49), anisotropic, hyperplastic materials, using the two-fiber family Holzapfel-Gasser-Ogden (HGO) material model with collagen fiber dispersion (see supplementary data) [24]. The region-specific HGO model parameters for the aortic wall were derived by fitting the HGO model to experimental biaxial stress-stretch data for the ASC,DSC,SAB, and IAB from Set 2 mice (Fig. 1H), following the protocol outlined in our previously published study [11]. Age, sex, and genotype-specific average material parameters were used in the FSI simulations (Supplementary Table S1A–D). Representative fitting results for each aortic region are presented in Supplementary Fig. S2. Age, sex, and genotype-specific incremental moduli corresponding to diastolic pressure from Set 2 mice were used as the elasticity moduli for mesh stiffness initialization (Supplementary Table S2) [9–11].

#### 2.5.4. Boundary conditions

##### 2.5.4.1. Fluid domain.

Inlet and outlet boundary conditions for the fluid domain for each simulation were defined using MR flow rate data (Fig. 1D) and a three-element Windkessel model (R-C-r) (Fig. 1E). In the Windkessel model, R and r represent the proximal and distal resistances for blood flow at the outlet arteries, respectively, while C denotes the compliance of the outlet arteries. The outlet boundary conditions are important for incorporating the downstream vasculature effects during FSI simulation. The inlet flow waveform was derived using MR-measured velocity and cross-sectional area (Supplementary Table S3A–B). The flow waveform was Fourier interpolated using 20 nodes. For seven of the 1 mo mice (2 WT males, 2 WT females, 2 MU males, and 1 MU female), the velocity data were deemed unreliable. Therefore, velocity values from age, sex, and genotype-matched mice were used to obtain the flow data. The mouse specific R-C-r parameters (Fig. 1E) at outlets 1, 2, 3, 4 and 5 (Fig. 1C) were tuned corresponding to the flow rate and blood pressure using the pressure-flow relationship (Eq. (2)),

Eq.2 $$\frac{\partial p}{\partial t}+\frac{p}{rC}=\frac{Q}{C}\left(1+\frac{R}{r}\right)+R\frac{\partial Q}{\partial t},$$

where p is the spatially averaged blood pressure at each outlet and Q is the flow rate at each outlet. Eq. (2) was iteratively solved over multiple cardiac cycles and tuning was considered successful when systolic blood pressure, diastolic pressure, mean arterial pressure, and pressure amplitude were all within a 5% tolerance range. Systolic and diastolic pressures were measured in Set 2 mice (Fig. 1I) [9] and age, sex, and genotype-matched average pressure values (Supplementary Table S4) were used to tune the R-C-r boundary conditions in the Windkessel model. The resulting values of R-C-r for each FSI simulation for Set 1 mice are given in Supplementary Table S5A–5D.

##### 2.5.4.2. Solid domain.

For the solid domain, Dirichlet-type fixed boundary conditions were applied at the inlet and outlet surfaces. The effect of surrounding tissues was incorporated by imposing a Robin type boundary condition on the aortic outer wall [25] (Eq. (3)) to apply traction normal to the aortic wall surface,

Eq.3 $$t_{s,n}=-k_{s}u-c_{s}\frac{\partial u}{\partial t}-p_{0}n,$$

where $t_{s,n}$ is the traction normal to the surface of the outer aortic wall due to reaction of surrounding tissues, n is a unit vector normal to the aortic outer wall surface, u is the local tissue displacement, ∂u/∂t is the local tissue velocity, $k_{s}$ and $c_{s}$ are the stiffness and viscous damping of surrounding tissues, and $p_{0}$ is the external pressure of the abdominal and thoracic aortic cavities, which was assumed to be zero during the simulation. The values of $k_{s}$ and $c_{s}$ were chosen as 1.0^3^ Pa mm^−1^ and 0.1 Pa s mm^−1^, respectively. These values were in the range reported in the literature [25,26].

#### 2.5.5. Prestress on the solid domain

The geometric model of the aorta, derived from MR imaging, represents a loaded or prestressed geometry corresponding to the diastolic blood pressure. Neglecting this loaded or prestressed state will lead to inaccuracies in simulating aortic deformation. Additionally, previous studies showed that including a prestress helps to reduce numerical artifacts such as deviations in aortic diameter and drops in diastolic pressure [16,27]. Thus, we adopted a previously established method to compute the prestress in the aortic wall corresponding to the diastolic state [16]. First, a computational fluid dynamics (CFD) simulation was conducted in the fluid domain assuming a rigid wall with the measured inlet flow rates and tuned outlet R-C-r boundary conditions. The simulation was run for 8–10 cardiac cycles, which were sufficient to achieve steady diastolic and systolic pressure and velocity profiles. From this simulation, the fluid-wall traction vectors were extracted and used as the Neumann boundary condition on the luminal surface of the solid domain for finite element (FE) simulations, incorporating regional material properties of the aortic wall (Supplementary Table S1A–D). The prestress tensor was computed by ensuring mechanical equilibrium between the fluid traction and the solid domain [16,28].

#### 2.5.6. FSI post processing

Once the prestress tensor was determined, the full FSI simulation was performed using the high-performance computing platform with 100 cores (Minnesota Supercomputer Institute and/or Washington University McKelvey School of Engineering Compute Cluster). The pressure and flow in the FSI simulation were initialized using the diastolic pressure and velocity values obtained from the CFD simulation corresponding to the last cardiac cycle. This reduced the number of cardiac cycles to obtain steady values of pressure and velocity and thus reduced overall computational time. The FSI simulation was performed for two cardiac cycles with a 0.0027 step time using the svFSI solver from SimVascular [21].

All post-processing analyses of the FSI simulation results were performed using ParaView and custom written Python scripts. FSI-derived metrics for the ASC, including wall shear stress related matrices such as oscillatory shear index (OSI), time averaged wall shear stress (TAWSS), endothelial cell activation potential (ECAP), and relative residence time (RRT) were calculated as defined in Eqs. 4a–d,

Eq. 4a $$\text{TAWSS}=\frac{1}{T}\int_{0}^{T}|\tau |dt,$$

Eq. 4b $$OSI=\frac{1}{2}\left(1-\frac{\left|\int_{0}^{T}\tau dt\right|}{\int_{0}^{T}|\tau |dt}\right),$$

Eq. 4c $$\text{ECAP}=\frac{OSI}{\text{TAWSS}},$$

Eq. 4d $$\text{RRT}=\frac{1}{\left(1-2\times \text{OSI}\right)\times \text{TAWSS}},$$

where T is total time of one cardiac cycle, and |τ| is the magnitude of wall shear stress τ. TAWSS is the time averaged wall shear stress over the full cardiac cycle. OSI indicates the change in direction of the wall shear stress vector. ECAP and RRT are metrics designed to identify the combined effect of changes in wall shear stress direction and magnitude and indicate endothelial sensitivity [29] and state of degraded flow [30], respectively. Intramural, von Mises, stress was also calculated. All analyses were specifically focused on the ASC region, as the critical region for TAA progression and failure.

## 3. Statistical analysis

### 3.1. Linear mixed effects modeling with three-way ANOVA

Statistical analyses and plot generation were conducted using R-studio (Posit, PBC) and Prism (GraphPad 10.60.1). The longitudinal changes in aortic geometry (ASC diameter and AER) and FSI-derived metrics were analyzed using a linear mixed model (LMM) approach with the lme4 R-package [31,32]. In this model, age, sex, and genotype were considered fixed effects. Five MU mice (3 males and 2 females) died at different ages during the study period. Accordingly, in the LMM, age was treated as a categorical variable, and mouse ID was included as a random effect to handle unbalanced data and to account for repeated measurements obtained from the same mouse. Three-way ANOVA was applied to test the main and interaction effects of fixed variables age, sex, and genotype. The F value (variation in outcome attributed to each fixed effect) and significant p-values are reported. Post hoc analysis was conducted by computing estimated marginal means and pairwise contrasts to identify specific group differences, focusing on comparisons between genotypes within each sex and age, as well as between sexes within each genotype and age.

### 3.2. Correlation analyses

Geometric measurements, FSI-derived metrics, elastic fiber porosity and medial collagen content data did not follow a normal distribution; therefore, a nonparametric Spearman’s rank correlation test [33] was performed to assess the correlation of FSI-derived metrics and AER with ECM remodeling and MR-measured maximum diastolic inner diameter of the ASC, as a measure of TAA progression. The Spearman’s correlation coefficient (r) and p value were computed individually for each group (WT males, MU males, WT females, and MU females) and are presented as scatter plots. Linear trend lines were fitted using ordinary least squares regression for visualization purposes only, to illustrate the general direction of the relationship identified by the nonparametric Spearman correlation analysis.

5/11 MU mice died before the study endpoint and the rest were sacrificed at 4 mo to collect Set 2 data. To assess the relationship between geometric and FSI-derived metrics with mouse lifespan, as a measure of TAA outcomes, the correlation analysis was censored at a maximum age of 4 months [7] using the Tobit model [34]. Furthermore, to account for the expected changes in geometric and FSI-derived metrics with growth and maturation of the mouse between 1 and 4 mo [9, 11] the MU values were normalized to the average age and sex-specific WT values. The goodness of correlation between normalized geometric and FSI-derived metrics with mouse lifespan was measured by the Pseudo ${\text{R}}^{2}$ value. All correlation analyses were performed in R-studio (Posit, PBC).

## 4. Results

### 4.1. Mouse lifespan

For Set 1 mice, none of the 6 WT mice died before 4 mo, while 5/11 MU mice died before 4 mo. Of the 5 MU mice that died before 4 mo, male MU mice died earlier (33, 65, and 71 days old) than female MU mice (100 and 107 days old). We performed autopsies on the mice when possible and observed signs of aortic rupture (Supplementary Fig. S5). These results are consistent with our previously published data for Set 2 mice [9] and show sex-specific effects of lifespan in MU mice. Data are included for each mouse in Set 1 up to the maximum age that MR imaging was obtained.

### 4.2. ASC maximum inner diastolic diameter and AER

**Fig. 2.**
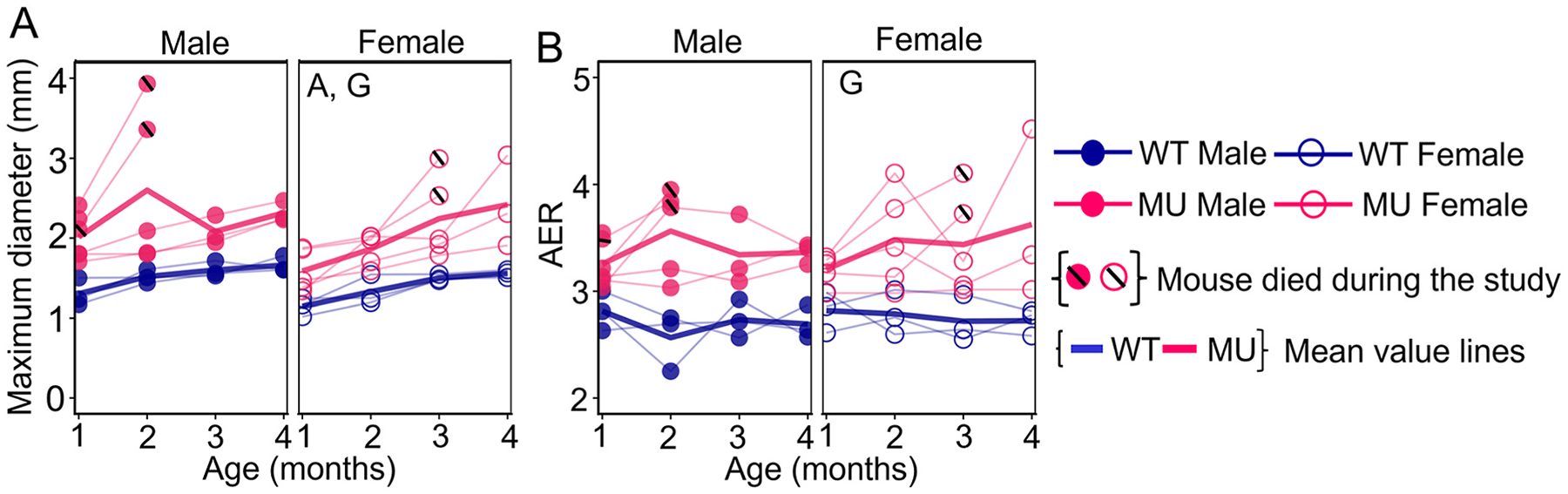
MR measured maximum inner diastolic diameter of ASC (A) and aortic elongation ratio (AER) (B). Letters in each panel indicate significant effects of the independent variables (A, age; S, sex; and G, genotype) by three-way ANOVA. Additional statistics are presented in Supplementary Table S6. Individual data points are shown and connected across time points for each mouse. Average values for each time point are also shown. For mice that died before 4 months of age (mo), a black line is shown in the symbol for the last recorded data point before death.

ASC maximum inner diastolic diameters for Set 1 mice are shown in Fig. 2A. Pairwise contrasts revealed significantly greater ASC diameter in MU males compared to WT males at all time points, whereas MU females exhibited increases in ASC diameter compared to WT females only at 3 and 4 mo (Supplementary Table S6A). MU males showed larger diameters than MU females at 2 mo (Supplementary Table S6A). Three-way ANOVA showed an effect of age (F=13.01,p<0.0001) and genotype (F=17.62,p=0.0015), indicating progressive dilation with age and larger diameters in MU mice compared to WT. The percentage differences in ASC diameter between MU and WT mice for each group are given in Table 1. Both male and female MU mice with large ASC diameters died before 4 mo. The last MR imaging timepoint before death is indicated by black lines across the circular symbols in Fig. 2A (1 male MU at 1 mo; 2 male MU at 2 mo; 2 female MU at 3 mo).

AER for MU and WT male and female mice in Set 1 are shown in Fig. 2B. AER increased significantly in male MU mice compared to WT at 2 and 3 mo and in female MU mice compared to WT at 2, 3, and 4 mo (Supplementary Table S6B). There were no significant differences in AER between males and females (Supplementary Table S6B). AER was significantly influenced by genotype (F=23.43,p=0.0004). The percentage differences in AER between MU and WT mice are given in Table 1. At 1 and 2 months of age, MU males with close to the highest AER did not survive to the next imaging timepoint. At 3 months of age, MU females with the highest AER did not survive to the next imaging timepoint.

### 4.3. ECM remodeling

**Fig. 3.**
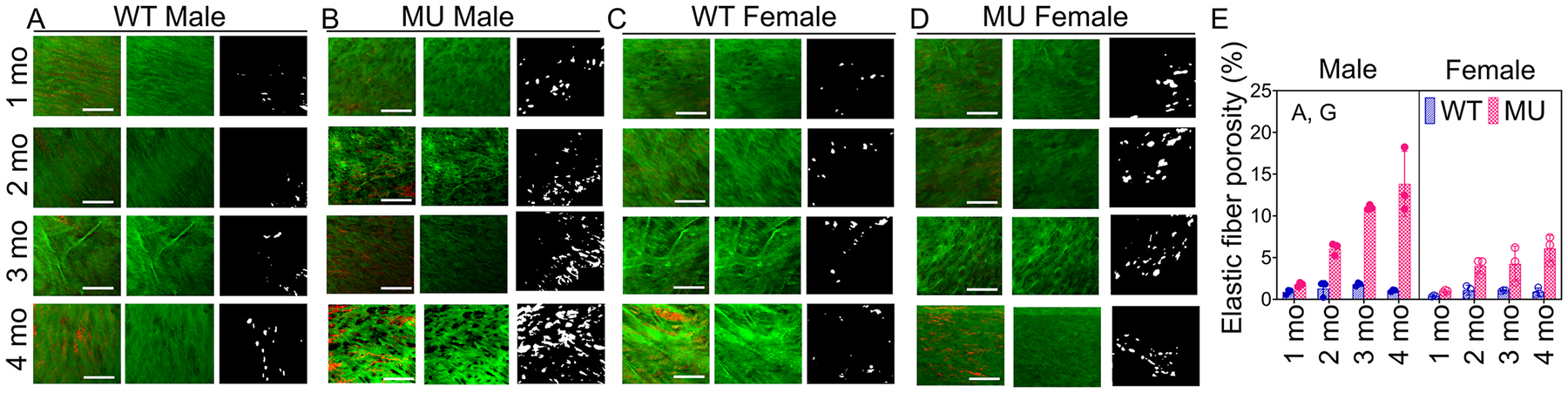
Representative en face multiphoton images of WT male (A), MU male (B), WT female (C) and MU female (D) ASC. The first column for each age, sex and genotype shows the collagen (red) and elastin (green) signals. The second column for each age, sex and genotype shows the elastin signal only, which was then thresholded to obtain the images in the third column. The scale bar in each image is 50μm. The thresholded images were used to determine the percent area of white pixels as a measure of elastic fiber porosity (E). Letters in panel E indicate significant effects of independent variables (A, age; S, sex; and G, genotype) and their interactions. Additional statistics are presented in Supplementary Table S7.

Representative en face multiphoton images of MU and WT male and female ASC at 1, 2, 3 and 4 mo from Set 2 mice are shown in Fig. 3A–D. Red (collagen) and green (elastin) signals are shown together in the first column for each sex and genotype. Collagen is only visible in MU ASC at later ages. Green (elastin) signal alone is shown in the second column for each sex and genotype. Visual inspection indicates age-dependent loss of elastic fibers that is more severe in male MU than female MU ASC. Elastic fiber porosity was quantitatively assessed by measuring the percent area of the white region after thresholding the images (third column for each sex and genotype). Fig. 3E shows the elastic fiber porosity at each age in MU and WT male and female mice. Both age (F=4.04,p=0.033) and genotype (F=9.43,p=0.015) had significant effects on elastic fiber porosity. Pairwise comparisons showed significant increases in elastic fiber porosity for MU males at 3 and 4 mo compared to WT and no significant differences between MU and WT female mice at any age (Supplementary Table S7). Medial collagen fiber content from Set 2 mice was determined from our previously published work [9] and was only used for correlation analysis (Section 4.6).

### 4.4. FSI-derived metrics

**Fig. 4.**
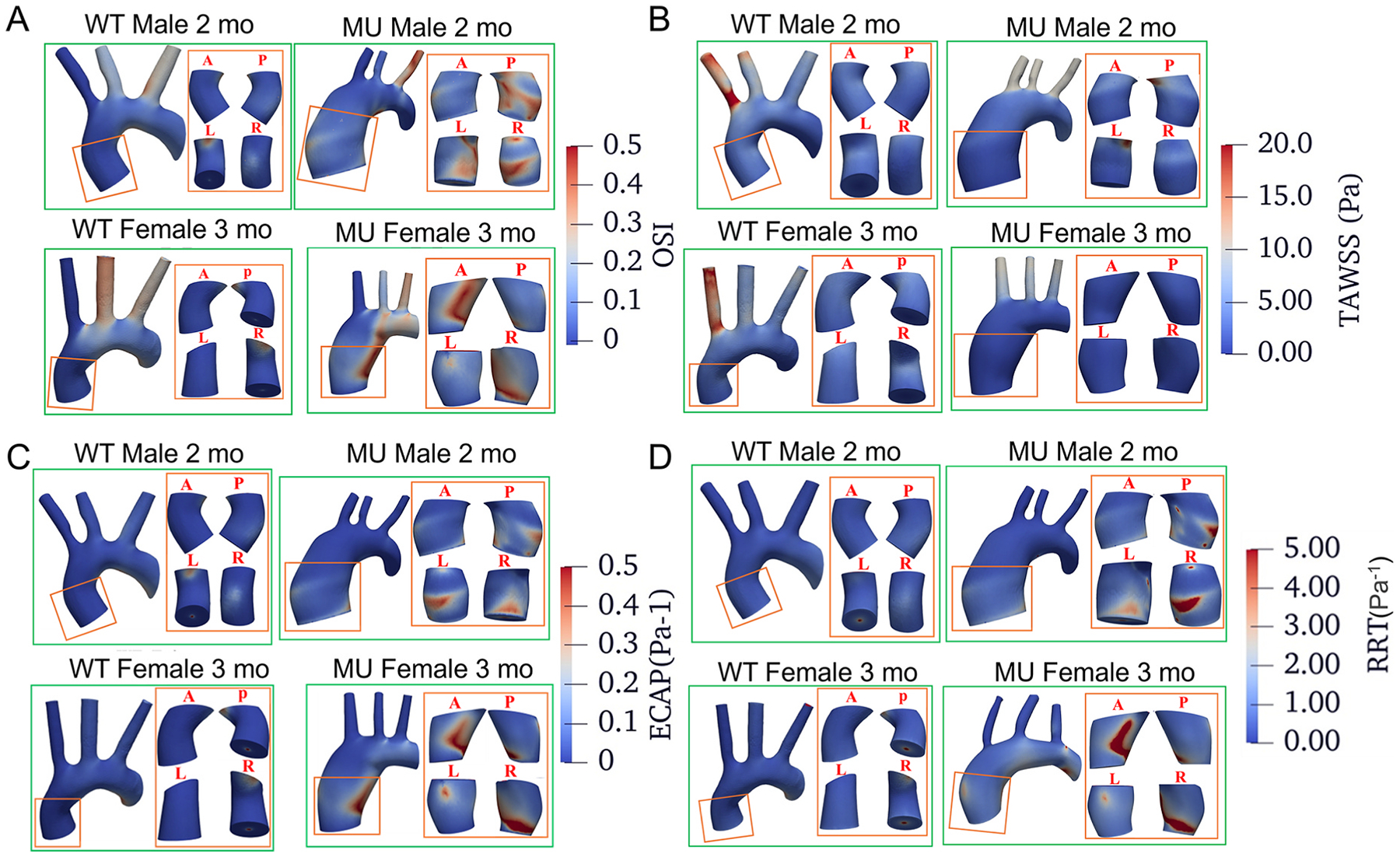
Maps of hemodynamic metrics over the ASC for OSI (A), TAWSS (B), ECAP (C) and RRT (D) for one of the MU male and female mice that died before the 4 mo study endpoint and representative WT mice at the same age. The ASC area in the red box is reproduced with four different views, anterior (A), posterior (P), right (R), and left (L), in the subpanels. Maps of hemodynamics metrics over the entire aortic length are presented in Supplementary Figs. S2–S3.

Although FSI simulations were performed on the entire aorta, our analysis was specifically focused on the ASC, as this region is particularly susceptible to TAA development in MFS. From the FSI simulations, we extracted wall shear stress-related metrics, including OSI, TAWSS, ECAP, and RRT, as well as intramural stresses, including von Mises stress at systolic pressure. All results are presented corresponding to the last simulation cycle. Maps of wall shear stress-related metrics in the ASC are shown for two of the MU male and female mice that died before the 4 mo timepoint and for representative WT male and female mice at corresponding ages (Fig. 4). MU ASC have areas of increased OSI (Fig. 4A), ECAP (Fig. 4C), and RRT (Fig. 4D). Maps of the wall shear-stress related metrics and the systolic von Mises stresses for the same mice for the entire aorta are provided in Supplementary Figs. S3–4.

**Fig. 5.**
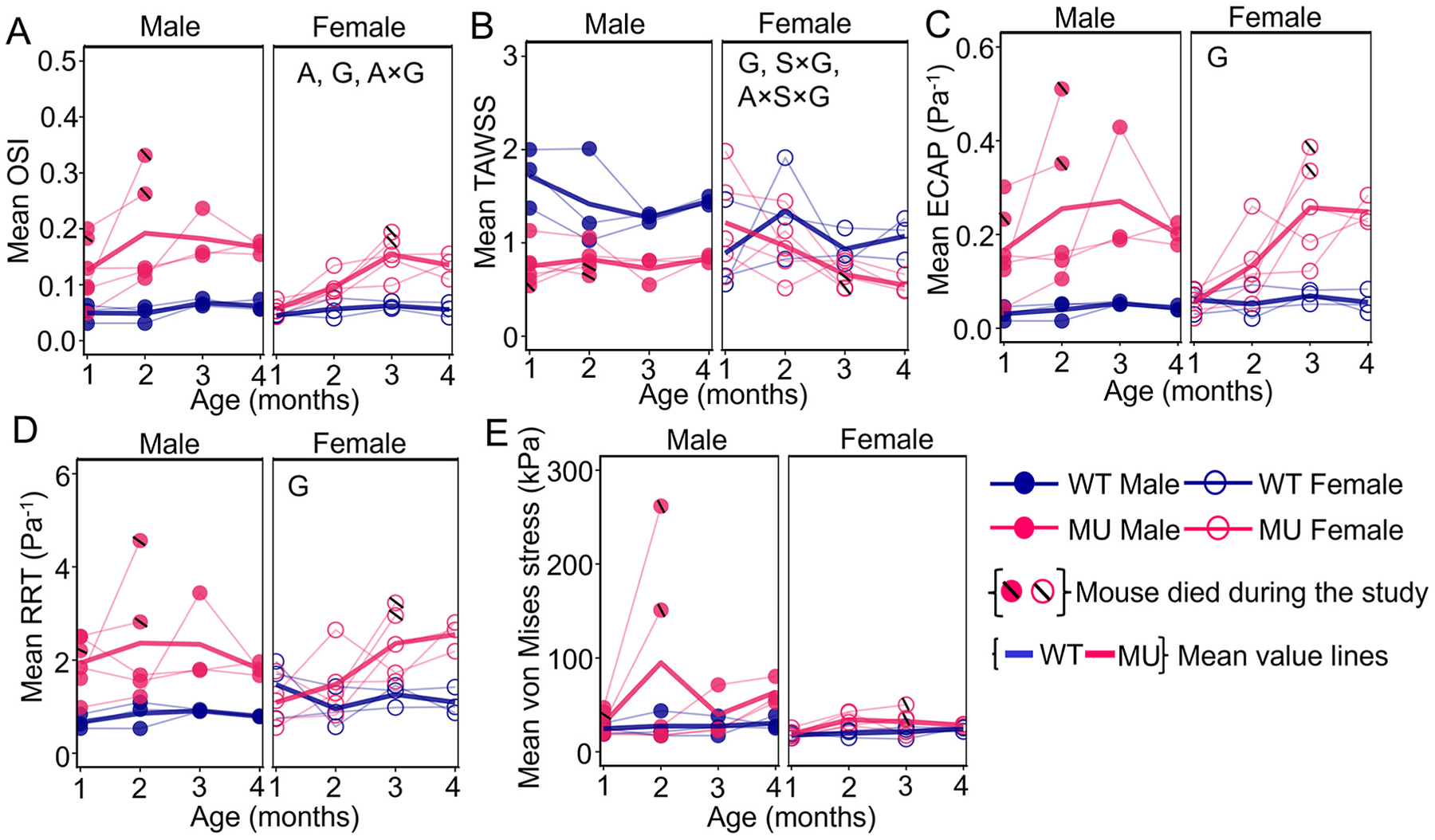
FSI-derived metrics for the ASC including mean OSI (A), mean TAWSS (B), mean ECAP (C), mean RRT (D) and mean systolic von Mises stress (E). Letters in each panel indicate significant effects of independent variables (A, age; S, sex; and G, genotype) and their interactions by three way ANOVA. Additional statistics are presented in Supplementary Table S8. Individual data points are shown and connected across time points for each mouse. Average values for each time point are also shown. For mice that died before 4 months of age (mo), a black line is shown in the symbol for the last recorded data point before death.

Individual and mean shear stress-related metrics and von Mises stresses for the ASC are quantified in Fig. 5 with posthoc pairwise analyses in Supplementary Table S8A–E. MU male ASC exhibited significantly higher mean OSI values than WT male ASC at all ages, whereas MU female ASC demonstrated significantly higher OSI compared to WT female ASC only at 3 and 4 mo (Fig. 5A–Supplementary Table S8A). WT mice showed no significant sex-dependent differences in OSI at any age. In contrast, MU mice exhibited significant sex differences at early ages (1 and 2 mo), where males had higher OSI values than females. Three-way ANOVA showed significant effects of age (F=6.782,p=0.0018), genotype (F=30.70,p=0.001), and interactions between age and genotype (F=3.29,p=0.038) for mean OSI. The MU mice that died before the 4 mo study endpoint had close to the highest mean OSI values for each age and sex.

MU male ASC showed significantly lower mean TAWSS compared to WT at all ages, whereas MU female ASC showed a significant reduction in TAWSS compared to WT only at 4 mo (Fig. 5B–Supplementary Table S8B). ANOVA results showed that mean TAWSS was significantly affected by genotype (F=23.40,p=0.0004), the interaction between sex and genotype (F=6.57,p=0.024), and the interaction between age, sex, and genotype (F=3.15,p=0.0376). The MU mice that died before the 4 mo study endpoint had the lowest mean TAWSS values for each age and sex.

ECAP assesses the combined effect of changes in shear stress direction and magnitude during blood flow and their contribution to aortic events [29]. The changes in mean ECAP between MU and WT mice, as well as between male and female mice, followed a trend similar to that observed for mean OSI (Fig. 5C–Supplementary Table S8C). ANOVA results showed that mean ECAP was significantly affected by genotype only (F=46.53,p<0.0001). The MU mice that died before the 4 mo study endpoint had close to the highest mean ECAP values for each age and sex.

RRT is another parameter used to assess the combined effects of changes in shear stress direction and magnitude during blood flow and reflects the duration that blood remains near the aortic wall [30]. Male MU ASC showed significantly higher RRT compared to WT at each age, but female MU ASC showed significantly higher RRT only at 3 and 4 mo (Fig. 5D–Supplementary Table S8D). At 1 and 2 mo, RRT was significantly higher in MU male than MU female ASC. Mean RRT was significantly affected by genotype (F=32.10,p<0.0001). The MU mice that died before the 4 mo study endpoint had close to the highest mean RRT values for each age and sex.

The mean systolic von Mises stress values for MU and WT male and female ASC at 1, 2, 3, and 4 mo are presented in Fig. 5E. Male MU ASC had significantly higher von Mises stress than male WT ASC at 2 and 4 mo (Supplementary Table S8E). The von Mises stress was not affected by age, sex, genotype, or their interactions by three-way ANOVA. The MU mice that died before the 4 mo study endpoint had the highest mean von Mises stresses for each age and sex.

The percentage differences in FSI-derived metrics between MU and WT mice in each group are presented in Table 1. Overall, the FSI-derived metrics showed significant differences between MU and WT ASC and earlier changes in male MU ASC compared to female MU ASC. Typically, the MU mice with the most extreme (highest or lowest) FSI-derived metrics at each age died before the 4 mo study endpoint, suggesting that impaired hemodynamics contribute to worse TAA outcomes.

The reported p values reflect the number of mice available at each time point. Notably, despite a reduction in sample size at later ages, individual mouse trajectories exhibited a consistent direction of change in the measured metrics with disease progression.

### 4.5. Correlation analysis

**Fig. 6.**
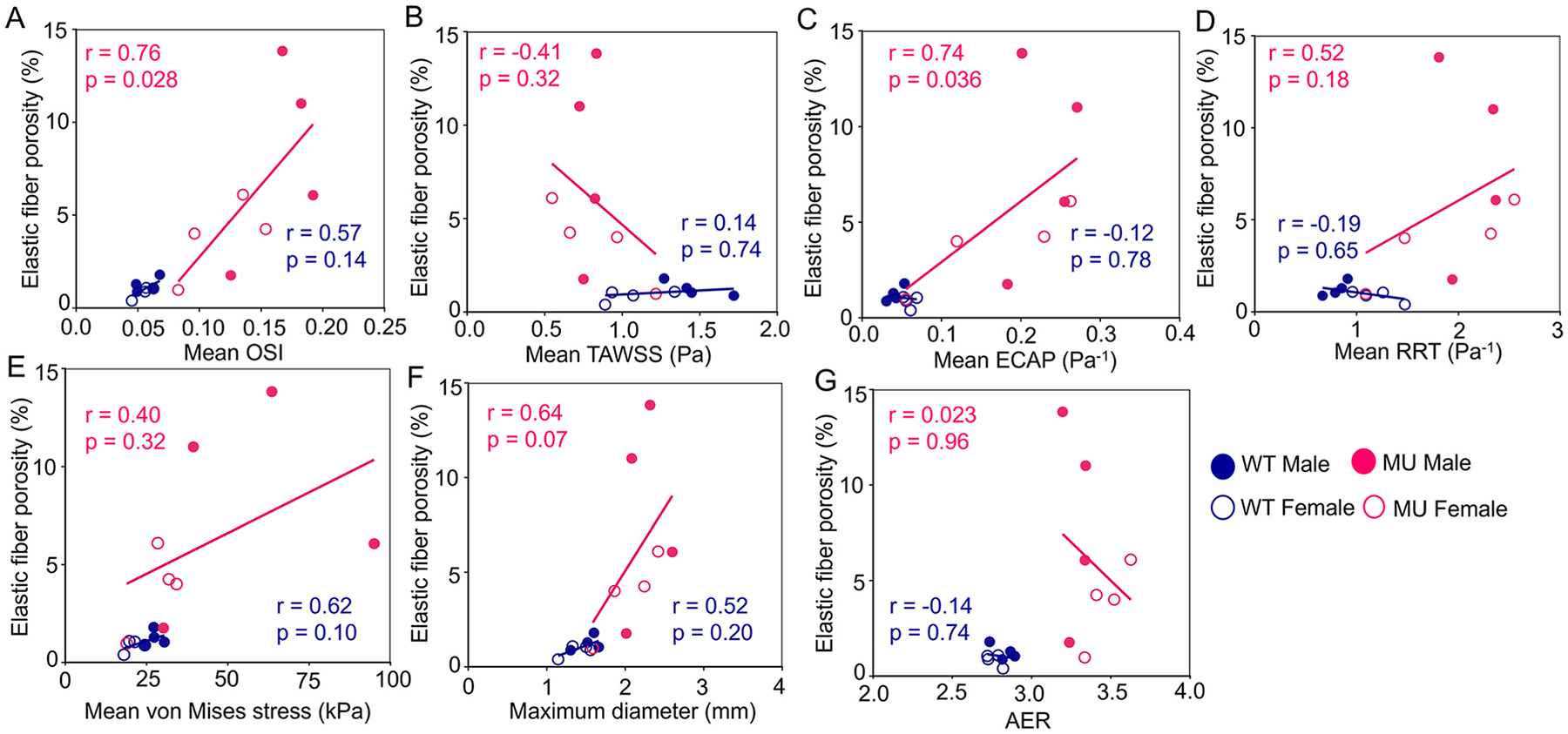
Spearman’s nonparametric correlation analysis between elastic fiber porosity and FSI-derived metrics, including mean OSI (A), TAWSS (B), ECAP (C), RRT (D), and systolic von Mises stress (E), and geometric metrics, including maximum inner diastolic diameter (F) and AER (G). The r and p values in each panel indicate the strength and significance of the correlation, respectively, for MU and WT aorta. The linear lines represent only the direction and trend of the correlations; they do not imply that the underlying relationship is strictly linear.

It has been hypothesized that hemodynamic changes affect remodeling of the aortic wall and may contribute to disease progression in TAA [19,20]. To investigate this hypothesis, we determined the correlation between geometric and FSI-derived metrics and elastic fiber porosity or medial collagen content for each genotype. Since data were collected from different sets of mice, average values for each, age, sex, and genotype were used for the correlation analysis with elastic fiber porosity and medial collagen fiber content. As expected with limited changes in ECM amounts and remodeling from 1 to 4 mo in WT ASC, there were no significant correlations for the WT data (Fig. 6 and Supplemental Fig. S6). For the MU ASC, there were significant correlations for 2 out of 7 of the elastic fiber porosity relationships (Fig. 6), but not for any of the medial collagen content relationships (Supplemental Fig. S6). The strongest correlations for the MU data were for mean OSI (r=0.76,p=0.028) (Fig. 6A) and mean ECAP (r=0.74,p=0.036) (Fig, 6C) with elastic fiber porosity. There were no significant correlations for the MU data for mean TAWSS (Fig. 6B), mean RRT (Fig. 6D), mean von Mises stress (Fig. 6E), maximum inner diastolic diameter (Fig. 6F), or AER (Fig. 6G) with elastic fiber porosity.

**Fig. 7.**
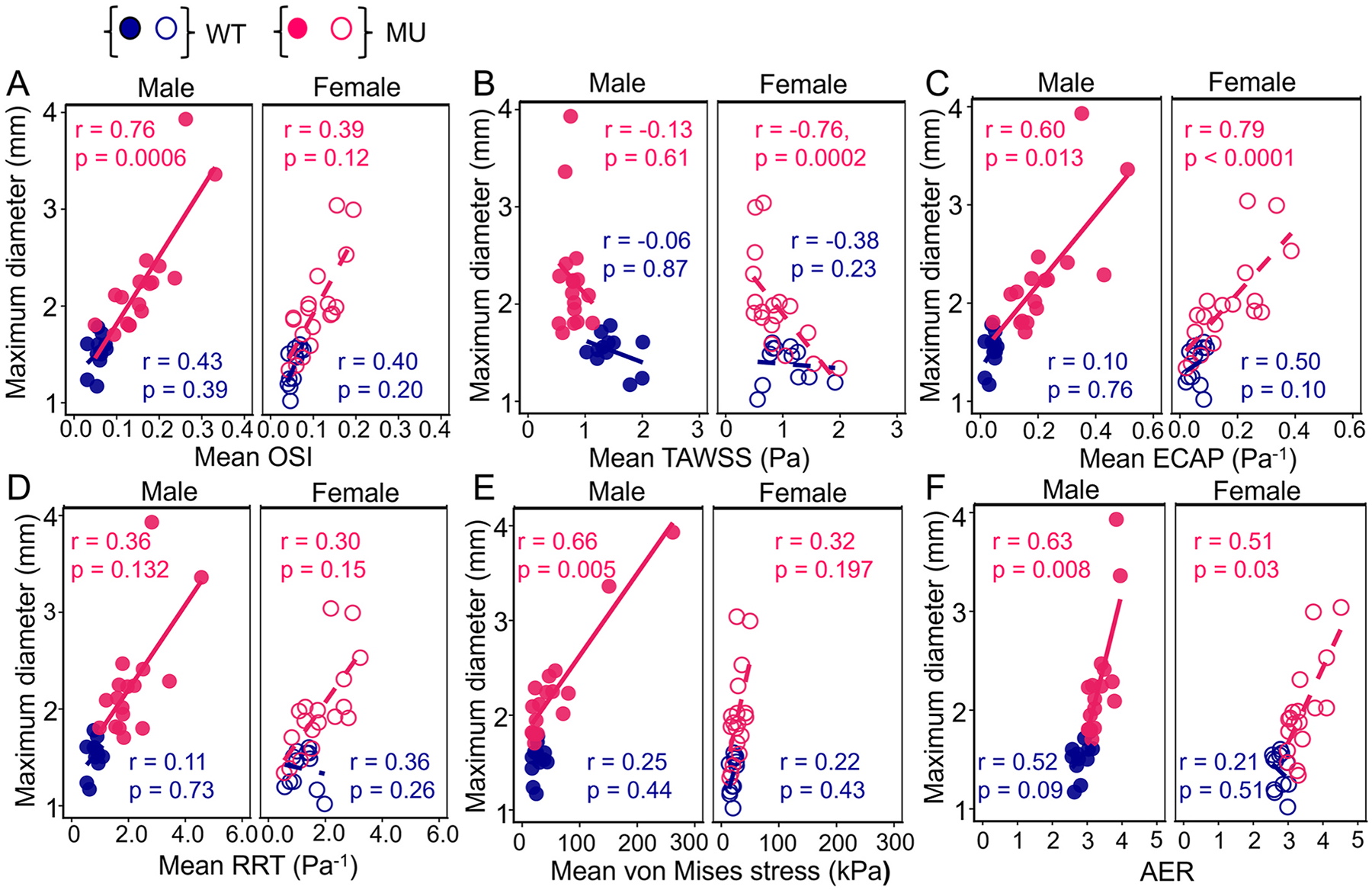
Sex and genotype specific Spearman’s nonparametric correlation analysis between maximum diastolic inner diameter and mean OSI (A), mean TAWSS (B), mean ECAP (C), mean RRT (D), mean systolic von Mises stress (E) and AER (F). The r and p values in each panel indicate the strength and significance of the correlation, respectively, for MU and WT male and female aorta. The linear lines represent only the direction and trend of the correlations; they do not imply that the underlying relationship is strictly linear.

Our overall goal was to determine metrics that can be used to gauge TAA progression and/or outcomes in male and female mice. Although diameter is not the best measure of TAA outcomes, it is the major clinical measure of progression and is used for surgical decision making. Hence, we investigated correlations between maximum inner diastolic ASC diameter and FSI-derived metrics and AER for MU and WT male and female mice (Fig. 7). For WT male and female mice, ASC diameter showed no significant correlations with any FSI-derived metrics or AER. In MU male ASC, OSI and mean von Mises stress showed a significant positive correlation with ASC diameter, whereas in MU females, OSI and mean von Mises stress were not significantly correlated with ASC diameter (Fig. 7A and E). In contrast, mean TAWSS was significantly negatively correlated with ASC diameter in MU females, but not in MU males (Fig. 7B). Additionally, both ECAP and AER were significantly positively correlated with ASC diameter in both male and female MU mice (Fig. 7C and E), while RRT showed no significant correlation with ASC diameter in either sex (Fig. 7D). These results indicate sex-dependent relationships between hemodynamics and TAA progression in MU mice. The linear lines in each figure panel only indicate the trends (positive or negative) of the relationships for visualization purposes.

**Fig. 8.**
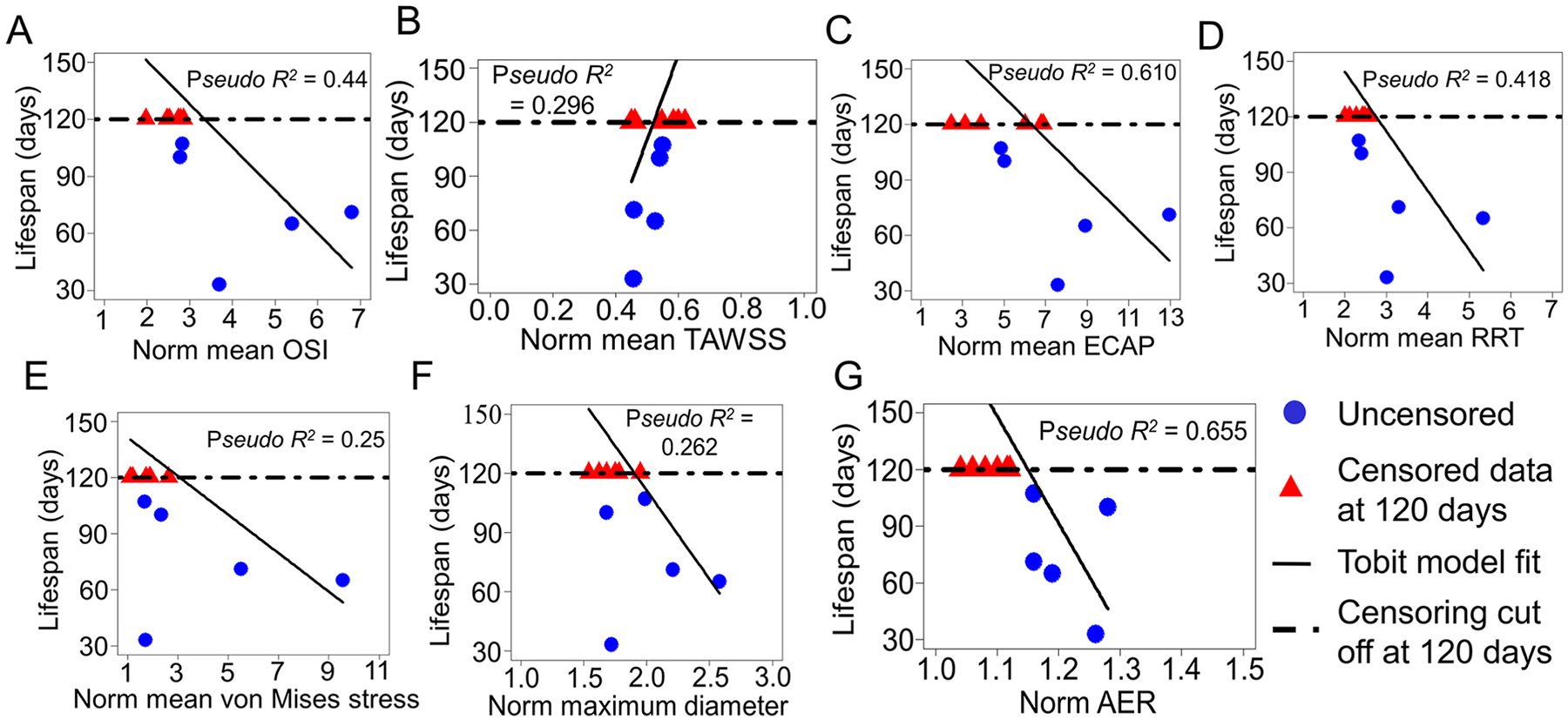
Tobit model analysis shows correlation between MU mouse lifespan and normalized mean OSI (A), mean TAWSS (B), mean ECAP (C), mean RRT (D), mean systolic von Mises stress (E), maximum diastolic inner diameter (F) and AER (G). The data were normalized to the WT values for the same age and sex and were censored at the study end point of 4 mo. Pseudo ${\text{R}}^{2}$ values indicate the strength of correlation. The linear lines represent only the direction and trend of the correlations; they do not imply that the underlying relationship is strictly linear.

For the MU mice only, we investigated the correlations between geometric and FSI-derived metrics and mouse lifespan as a measure of TAA outcomes (Fig. 8). The geometric and FSI-derived metrics are normalized to the average WT values for each age and sex. ECAP (*Pseudo* $R^{2}=0.610$) (Fig. 8C) and AER (*Pseudo* $R^{2}=0.655$) (Fig. 8G) emerged as the strongest predictors of MU mouse lifespan. Von Mises stress, which reflects intramural wall stress, and maximum inner diastolic ASC diameter, which is the major factor used to make clinical decisions in TAA, showed the weakest correlations with MU lifespan (Pseudo $R^{2}=0.25$ and 0.262, respectively).

## 5. Discussion

We used a severe mouse model of TAA associated with MFS $\left(Fbn1^{mgR/mgR}\right)$ to develop mouse-specific FSI simulations and investigate age- and sex-specific alterations in aortic hemodynamics and wall mechanical stresses. We correlated FSI-derived metrics with ECM remodeling, TAA progression, and outcomes. Our overall goal was to identify potential non-invasive biomarkers for improved TAA management.

### 5.1. Comparisons to previous work

A significant body of research has explored mouse aortic hemodynamics through computational modeling, but most of this work relies on rigid-wall CFD assumptions [35–39]. FSI simulations are necessary to properly reproduce complex hemodynamics in biological tissues with large deformations, such as the aorta [40]. There are some studies where mouse-specific three-dimensional FSI simulations have been used to assess hemodynamics of the aorta in applications such as aging [23,41], atherosclerosis [42,43], and abdominal aortic aneurysms [44].

With respect to mouse-specific FSI simulations for TAA, Bazzi et al. [15,22] developed models to predict biofluid biomarkers for TAA progression and outcomes in a genetic mouse model associated with mutations in fibulin-4. The results of Bazzi et al. are consistent with our observations that FSI-derived metrics are stronger predictors of TAA outcomes, such as mouse lifespan, than maximum aortic diameter. Although Bazzi et al. presented important findings, their models assumed identical flow conditions for all mice and uniform wall mechanical properties along the aortic length. However, aortic wall mechanical properties vary along the aortic length and are differentially affected by sex and genotype [10], which may influence overall hemodynamic responses [23]. The primary contribution of the present work is the investigation of age, sex and genotype-dependent variations in ECM remodeling, geometric changes, and FSI-derived metrics using comprehensive experimental data and the evaluation of their potential utility in TAA management.

### 5.2. FSI-derived metrics and their correlation with ECM remodeling

MU ASC of male and female mice showed altered hemodynamics, including increased OSI, ECAP, and RRT and decreased TAWSS, that progressively worsened with increased age (Fig. 5). OSI and TAWSS are independent hemodynamic measures that do not correlate with each other, while ECAP and RRT are derived measures that correlate with OSI and TAWSS (Supplementary Table S9), as expected by their mathematical definitions (Eq. (4)). ECAP and RRT were defined to capture the combination of high OSI and low TAWSS that locally affect endothelial cell mechanobiology [29,30] and correlate with regions of atherosclerotic plaque progression [45] and thrombus development in abdominal aortic aneurysms [46]. The same mechanobiological changes that stimulate plaque progression and thrombus development can promote ECM remodeling that contributes to growth of cerebral aneurysms [47] and rupture of abdominal aortic aneurysms [48]. In TAA, alterations in wall shear stress correlate with areas of elastic fiber degradation [19, 20].

We confirmed that elastic fiber porosity correlates significantly with OSI and ECAP in male and female MU mice (Fig. 6). We speculate that the Fbn1 mutation initially disrupts the microstructural integrity of the aortic wall, leading to changes in wall geometry. These geometric alterations subsequently cause abnormal blood flow, which in turn generates altered hemodynamic and biomechanical stresses [47]. These abnormal stresses activate mechanotransduction pathways, in the long term, leading to changes in cell phenotype, activation of inflammatory factors, and induction of cell apoptosis [49,50]. These changes further stimulate ECM degradation, alter aortic geometry, and promote TAA progression [51]. However, in MU mice, these changes are sex-specific with females showing partial protection from the pathologic feed-forward cycle of ECM remodeling and TAA progression [9]. Future work should focus on the mechanisms of protection in females to identify possible therapeutic targets.

Elastic fiber degradation in TAA is often associated with increased collagen fiber deposition [10]. We showed increased medial collagen fiber deposition in MU male ASC at 3 and 4 mo compared to WT [9], however medial collagen content did not correlate with FSI-derived or geometric metrics in the current study (Supplemental Fig. S6). This may be due to the more modest changes in collagen fiber deposition compared to elastic fiber degradation in mouse models of MFS that were observed in our work [9,10] and others [14,52].

### 5.3. Lifespan and aortic geometry

Aortic diameter is the metric currently used to determine the timing for clinical intervention in TAA. Our results indicate that MU mice with the largest ASC diastolic inner diameters at young ages do not survive to the study endpoint (Fig. 2A), supporting this clinical metric. Male MU mice had larger dilation and earlier death than female MU mice, consistent with previous studies [9,53,54]. However, female MU mice exhibited smaller increases in maximum inner diastolic diameter compared to WT prior to death (92 – 127%) than male MU mice (125 – 160%). Additionally, diameter did not correlate well with lifespan (Fig. 8F). These findings suggest the need for distinct, sex-specific diameter thresholds for effective TAA management, as well as additional criteria that are related to TAA outcomes.

Like maximum diameter, aortic length or AER can be measured directly from non-invasive CT and MR imaging [55]. In humans, ASC elongation occurs concurrently with dilation [56], precedes TAA dissection [57], and is associated with an increased rate of adverse aortic events [58]. The aorta elongates and can become tortuous with aging [59] and with non-syndromic [60] and syndromic TAA [61]. In mice, aortic lengthening and tortuosity are associated with defects in elastic fibers [62–66], hence AER may serve as a surrogate measure of elastic fiber degradation that contributes to TAA progression and failure. Our results showed higher AER in MU mice compared to WT at each age, with the highest AER values in male and female mice that died before the study endpoint (Fig. 2B). AER showed strong correlations with maximum aortic diameter (Fig. 7F) and reduced lifespan (Fig. 8G) in MU male and female mice, supporting its use as an additional criterion to diameter for clinical TAA management [55].

### 5.4. Identification of biophysical biomarkers for TAA progression and outcomes

Using maximum inner diastolic diameter as a proxy for TAA progression, the only FSI-derived metric that significantly correlated with progression in both male and female MU mice was mean ECAP (Fig. 7). These results support the feed-forward relationship hypothesized between hemodynamics, cellular mechanobiology, ECM remodeling, and TAA progression [47,49,51]. Interestingly, other FSI-derived metrics had significant correlations with TAA progression, but not in both sexes. For example, mean OSI and von Mises stress correlated significantly with maximum inner diastolic diameter in male MU mice only and mean TAWSS correlated significantly with maximum inner diastolic diameter in female MU mice only. While it may be expected that some FSI-derived metrics will correlate with diameter based on simplified analyses (i.e. shear stress would go down and circumferential stress would go up as diameter increases for steady, laminar flow in a straight tube), the sex dependent results suggest that these simplified analyses do not apply in the complex hemodynamic environment of unsteady flow in a deformable, curved tube. The sex-dependent differences in possible biophysical biomarkers may be due to variations in timelines or mechanisms of TAA progression in males and females that should be investigated in future work.

Using mouse lifespan as a proxy for TAA outcomes, ECAP also had the strongest correlation with TAA outcomes of all the FSI-derived metrics (Fig. 8C). These results support the premise that high OSI and low TAWSS that locally affect endothelial cell mechanobiology [29] contribute to adverse outcomes in multiple aortic diseases [45,46,48]. For clinical translation, ECAP can be determined through patient-specific computational modeling using CT or MR imaging to obtain the aortic geometry [67], as done in this study. However, data required for the computational model including inlet flow profiles, aortic material properties, surrounding tissue support, etc. are challenging to obtain in humans. 4D flow MRI has emerged as a tool to obtain patient-specific inlet flow profiles contributing to more realistic computational models [68]. 4D flow MRI can also be used to directly measure hemodynamic metrics [69], but has been limited by spatial resolution and image noise. 4D flow MRI combined with artificial intelligence trained super-resolution networks shows promise to advance full-field flow imaging [70] and provide clinical measures of metrics such as ECAP.

### 5.5. Limitations and future work

One major limitation of this study is the low sample size in Set 1 due to the aggressive disease phenotype leading to early death in MU mice and the resource intensive nature of longitudinal MR imaging and computational modeling of individual mice. Low sample size can lead to overinterpretation of the results, hence future work should include additional studies in more mice, as well as in other mouse models of MFS that have a longer lifespan. However, the consistency of our results with other available studies supports our conclusions. Although we aimed to perform mouse-specific FSI simulations and individual correlations, we could not collect all data for each mouse. Blood pressure, elastic fiber porosity, aortic wall thickness, and region-specific material properties were collected from Set 2 mice and average values for each age, sex, and genotype were used in the FSI simulations for Set 1 mice or the correlations between metrics (i.e. Fig. 6). Future research should include experimental and computational results that focus on individual mice and on detailed regional characterization (i.e. at the location of aortic rupture) to better understand and predict patient-specific TAA progression and outcomes.

In the FSI simulations, we assumed a uniform thickness (derived from ASC measurements) along the length of the aorta, which may affect the region-specific hemodynamics. However, the primary focus of this study was on the ASC, and the thickness assumptions are correct in this region. We assumed constant values for the stiffness and viscous damping parameters for the surrounding tissue support across age, sex, genotype and aortic region. A sensitivity analysis showed that large changes in the parameters had minimal effects on wall shear stress. A previous study showed that similar stiffness and damping parameters for the surrounding tissue were appropriate for healthy and diseased mouse aorta, provided changes in aortic wall stiffness were included [71], which we did. However, future studies could include region-specific wall thickness and age-, sex-, genotype-, and region-specific stiffness and damping parameters, which are especially important for estimating pulse wave velocity [23,72]. Additionally, although we included mouse-specific inlet flows, cardiomyopathy in MU mice [73] may further affect the hemodynamic results.

For geometric measures, we focused on maximum diameter and AER, as easily measurable quantities from noninvasive imaging. More sophisticated geometric measures, such as statistical shape analysis [74, 75], could be included in future work. We focused on the ASC for this study, but we have data on the entire aorta. Future research could explore hemodynamic alterations throughout the aortic length, as measurable dilation and ECM remodeling occur in other regions [10,13, 14,76]. We observed sex-specific differences in potential biophysical biomarkers, but we did not investigate the mechanisms for these differences. Lastly, our results in a severe mouse model of MFS should be tested in humans and in other TAA etiologies to determine the translational relevance and broader applicability of our conclusions.

## 6. Conclusions

We performed FSI simulations to assess age- and sex-specific changes in hemodynamics and aortic wall stresses and correlated the results with ECM remodeling, aortic dilation, and lifespan in a mouse model of severe MFS. The results suggest that abnormal hemodynamics play a role in TAA progression and outcomes, perhaps by promoting elastic fiber degradation in the aortic wall. Our findings reveal that geometric and FSI-derived metrics, specifically AER and ECAP, correlate with TAA progression and outcomes and may serve as additional biomarkers for personalized management of TAA in patients with MFS. These markers can be determined noninvasively through imaging and/or computational modeling and may be used in conjunction with sex-specific diameter thresholds to improve TAA care in men and women.

## Supplementary Material

1

## Figures and Tables

**Table 1:** The average percentage change for geometric metrics (maximum diameter and AER) and FSI-derived metrics in the ASC (mean OSI, TAWSS, ECAP, RRT, and systolic von Mises stress) between WT and MU mice for each age and sex.

	Male				Female			
Age	1 mo	2 mo	3 mo	4 mo	1 mo	2 mo	3 mo	4 mo
Maximum diameter (%)	54	71	30	39	38	40	50	55
AER (%)	15	30	22	23	18	23	25	28
OSI (%)	154	296	169	169	26	69	146	142
TAWSS (%)	−56	−42	−43	−42	37	−28	−29	−49
ECAP (%)	497	548	408	370	−9	130	233	371
RRT (%)	192	177	157	129	−26	54	84	133
von Mises stress (%)	23	248	45	109	5	74	49	17

## Appendix A. Supplementary data

Supplementary data to this article can be found online at https://doi.org/10.1016/j.compbiomed.2026.111594.
