# Supplementary material for: Alterations in ascending aortic hemodynamics and aortic length correlate with sex-specific thoracic aortic aneurysm dilation and lifespan in a mouse model of severe Marfan syndrome
Source: Comput Biol Med. Author manuscript; Available in PMC 2026 Apr 15. (PMC13081028; doi:10.1016/j.compbiomed.2026.111594)
Supplement: 1 [file NIHMS2163950-supplement-1.docx]

**Supplementary Data**

1. **Holzapfel-Gasser-Ogden (HGO) model**

The HGO model is given in Eq. S1,

| $\Psi=\frac{c}{2}\left( I_{1}-1 \right)+\sum_{i=1}^{2} \frac{k_{1}}{k_{2}}\left[ exp\left\{ k_{2}\left[ \kappa I_{1}+\left( 1-3\kappa\right)I_{4i}-1 \right]^{2}-1 \right\} \right]$. | Eq. S1 |
| --- | --- |

The first term of Eq. S1 is a Neo-Hookean solid and represents the ground matrix and elastic fiber contributions and the second term is a collection of two fiber families with exponential behavior and represents the anisotropic stiffening response of collagen fibers. In Eq. S1, *c* is an elastic modulus-like parameter, $I_{1}= \lambda_{\theta}^{2}+\lambda_{z}^{2}+\lambda_{r}^{2}$ is the first invariant, *k*_1_ > 0 is an elastic modulus-like parameter, *k*_2_ > 0 is a dimensionless parameter associated with material nonlinearity, $\kappa$ is a collagen fiber dispersion parameter ($0\leq\kappa\leq\frac{1}{3}$), the invariant $I_{4i}= \lambda_{\theta}^{2}{cos}^{2}\left( \alpha^{i} \right)+\lambda_{z}^{2}{sin}^{2}\left( \alpha^{i} \right)$ describes the stretch along the mean orientation of collagen fiber family *i* with angle *α*.


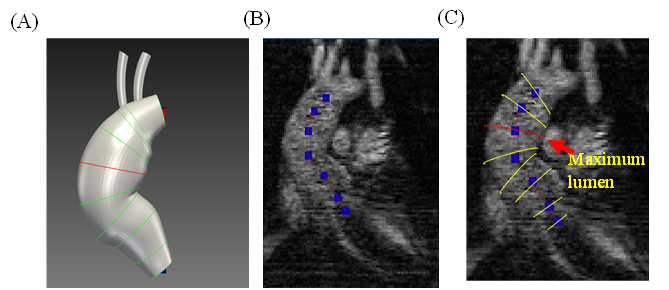


**Figure S1.** Methods for determining the maximum aortic diameter. (A) Segments of the aorta were identified from the root to the brachiocephalic artery. (B) The path and center line were extracted (red) and (C) the lumen diameter (yellow line) perpendicular to the center line was measured. The figures show a reduced number of diameter measurements for better visualization.

**
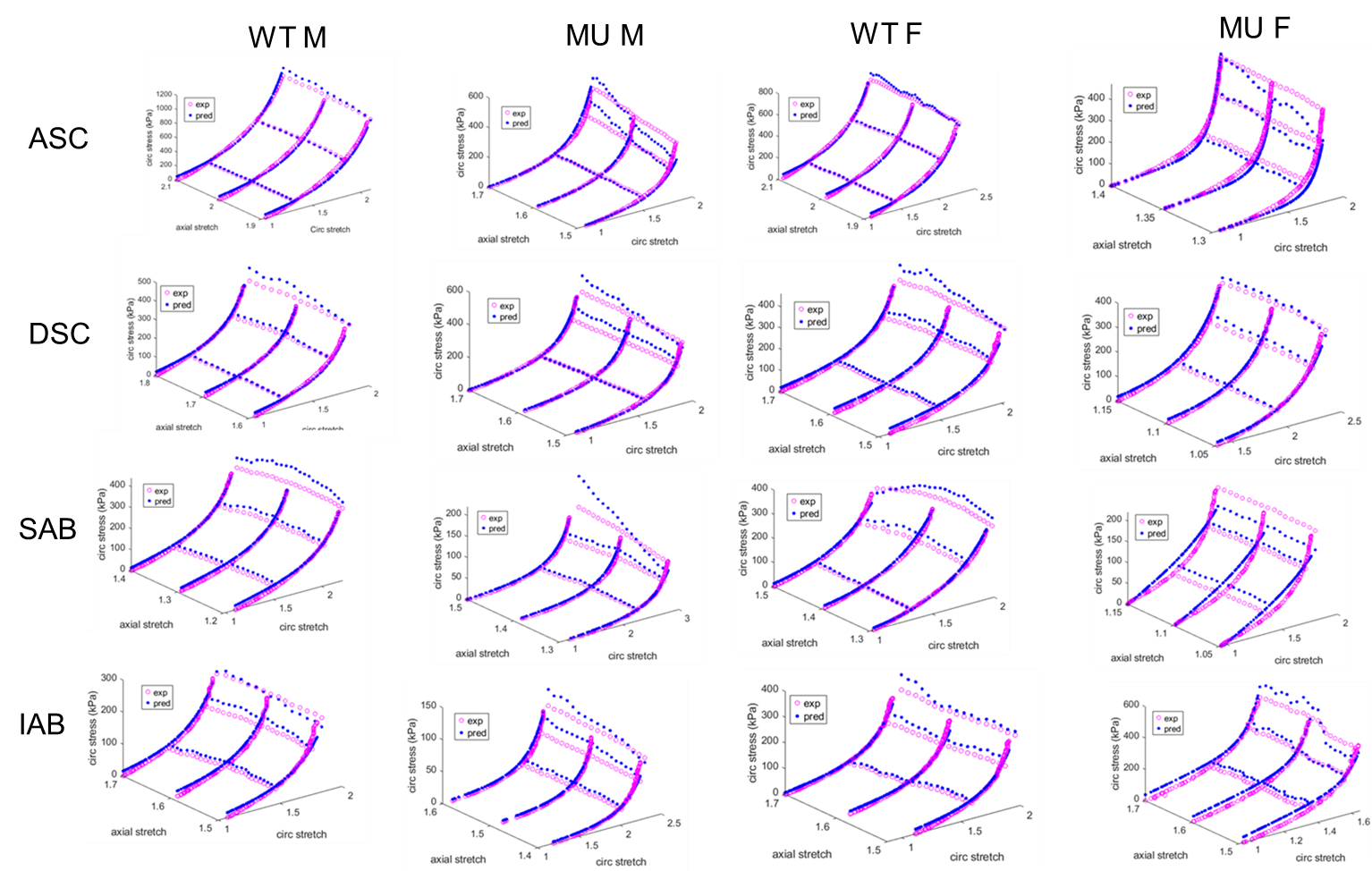
**

**Figure S2**. Representative results of the HGO model (Eq. S1) fitted to experimental stress-stretch data for each aortic region from 4 mo WT and MU male (M) and female (F) mice with the corresponding goodness-of-fit provided in Tables S1A–1D.


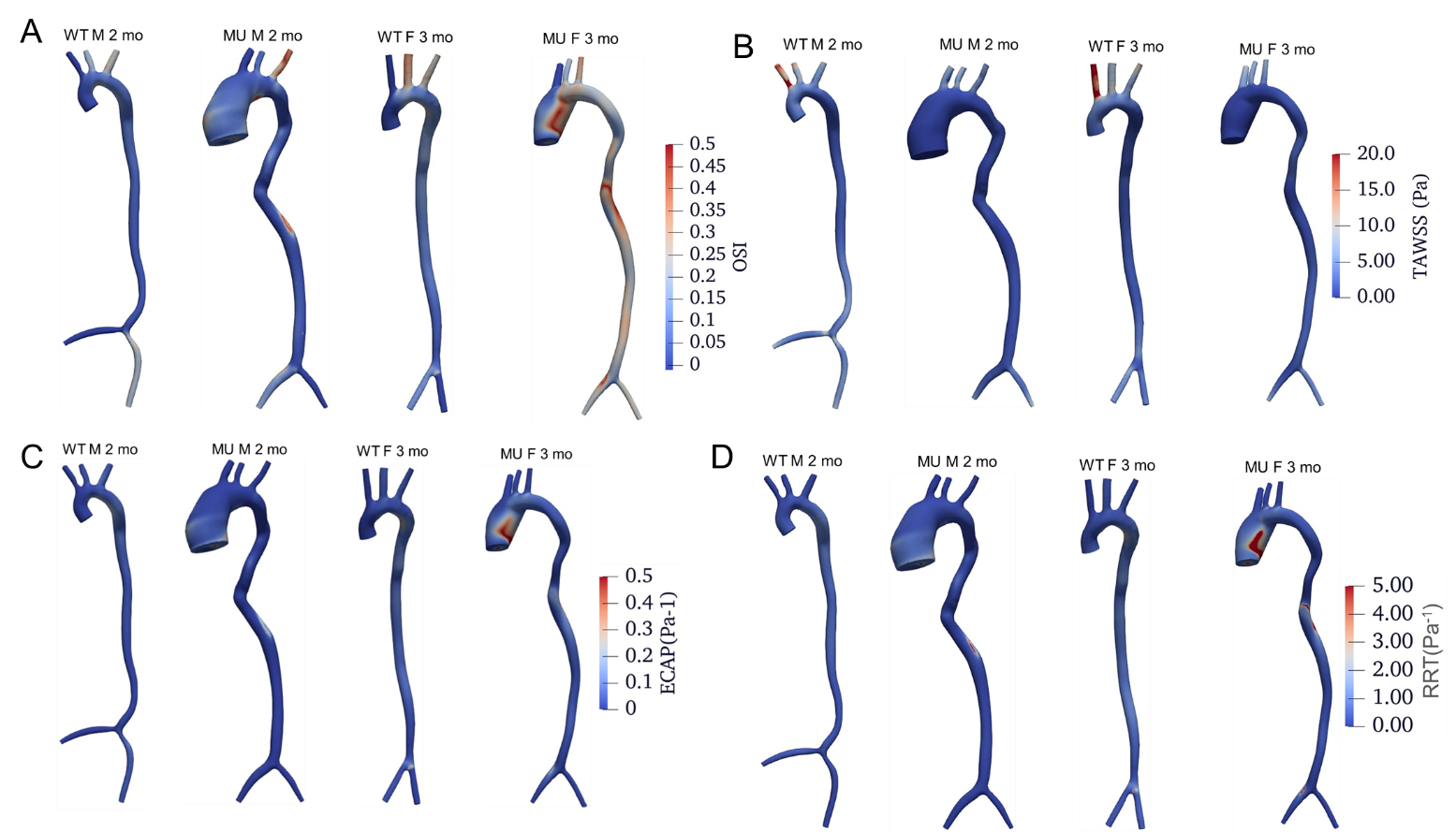


**Figure S3.** Map of FSI-derived parameters (A) OSI, (B) TAWSS, (C) ECAP and (D) RRT along the entire aorta for MU male (M) (2 mo) and female (F) (3 mo) mice that died before the 4 mo study endpoint and representative WT mice at equivalent ages.


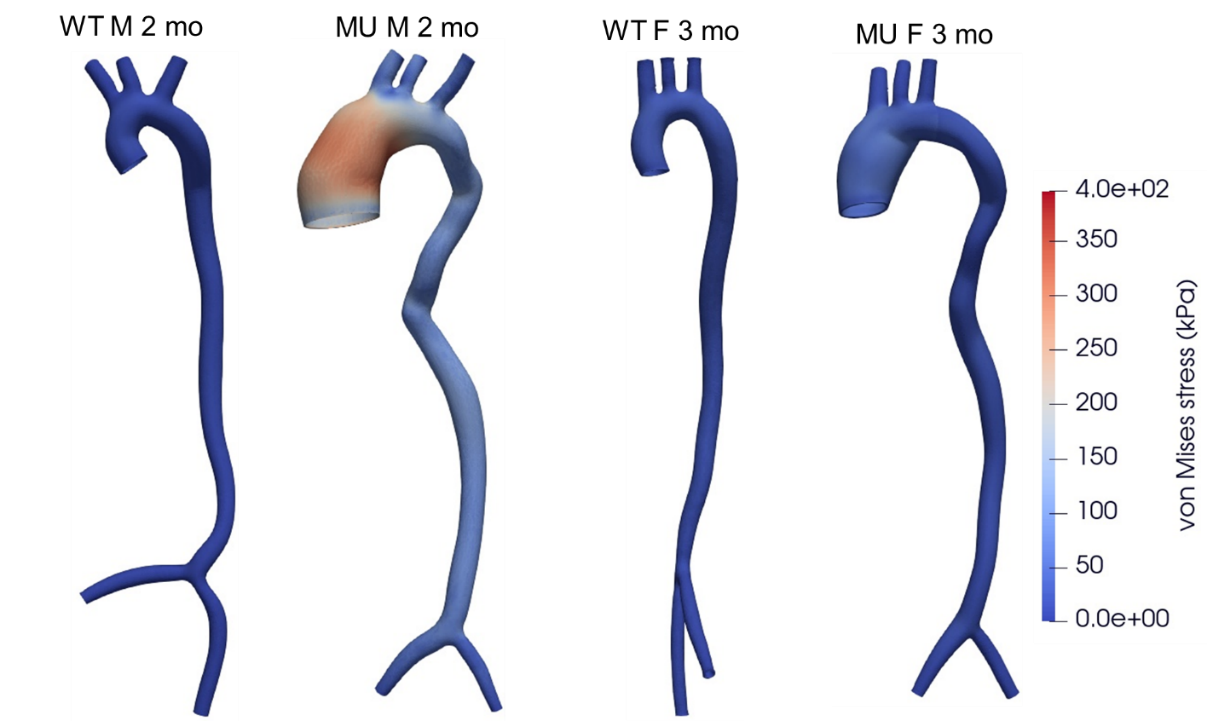


**Figure S4.** Map of systolic von Mises stress along entire aorta for MU male (M) (2 mo) and female (F) (3 mo) mice that died before the 4 mo study endpoint and representative WT mice at equivalent ages.


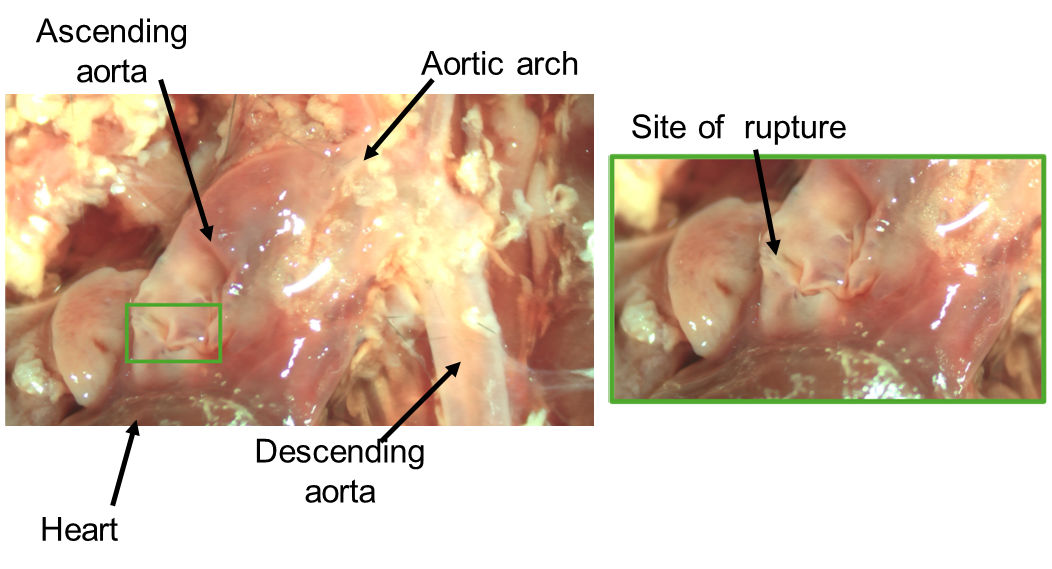


**Figure S5.** Image shows the location of rupture (enclosed by the green rectangular) near the aortic root in a MU male mouse that died at the age of 65 days.

**
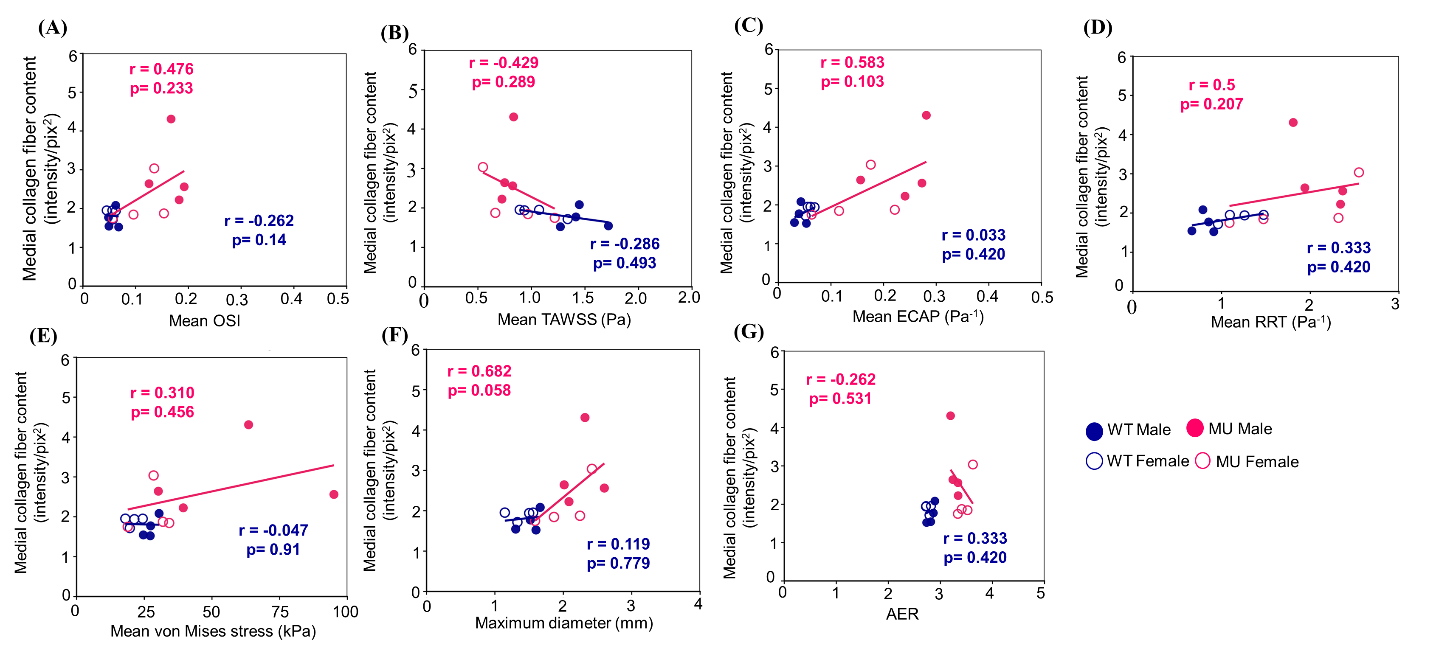
**

**Fig S6.** Spearman’s nonparametric correlation between medial collagen fiber content and FSI derived metrices, including mean OSI (A), TAWSS (B), ECAP (C), RRT (D), and systolic von Mises stress (E), and geometric matrices, including maximum inner diastolic diameter (F) and AER (G). The r and p values in each panel indicate the strength and significance of the correlation, respectively, for MU and WT aorta. The linear lines represent only the direction and trend of the correlation: they do not imply that the underlying relationship is strictly linear.

**Table S1A**. Average values of fitted HGO material parameters for ASC (N = 4 – 10/group). The R^2^ shows the goodness of fit. Experimental data came from [9-10]

| **ASC** | *WT Male* | | | | | |
| --- | --- | --- | --- | --- | --- | --- |
|  | ***c (kPa)*** | ***k_1_ (kPa)*** | ***k_2_*** | ***α (ᵒ)*** | ***κ*** | ***R^2^*** |
| 1 mo | 23 ± 12 | 17 ± 6 | 0.51 ± 0.36 | 12 ± 19 | 0.25 ± 0.09 | 0.84 ± 0.09 |
| 2 mo | 21 ± 8 | 19 ± 8 | 0.32 ± 0.15 | 20 ± 23 | 0.28 ± 0.04 | 0.85 ± 0.03 |
| 3 mo | 32 ± 17 | 17 ± 13 | 0.24 ± 0.14 | 32 ± 40 | 0.30 ± 0.02 | 0.80 ± 0.08 |
| 4 mo | 30 ± 6 | 14 ± 6 | 0.36 ± 0.26 | 9 ±17 | 0.30 ± 0.02 | 0.87 ± 0.03 |
|  | *MU Male* | | | | | |
| 1 mo | 9 ± 1 | 33 ± 26 | 1.00 ± 0.71 | 34 ± 37 | 0.17 ± 0.12 | 0.71 ± 0.11 |
| 2 mo | 12 ± 3 | 10 ± 4 | 0.73 ± 0.26 | 8 ± 14 | 0.23 ± 0.10 | 0.71 ± 0.06 |
| 3 mo | 17 ± 9 | 12 ± 6 | 1.19 ± 0.71 | 41 ± 37 | 0.25 ± 0.06 | 0.73 ± 0.07 |
| 4 mo | 20 ± 3 | 5 ± 4 | 0.87 ± 0.18 | 20 ± 20 | 0.14 ± 0.12 | 0.68 ± 0.06 |
|  | *WT Female* | | | | | |
| 1 mo | 27 ± 16 | 35 ± 8 | 1.72 ± 3.00 | 36 ± 44 | 0.28 ± 0.05 | 0.82 ± 0.07 |
| 2 mo | 19 ± 2 | 36 ± 6 | 0.49 ± 0.09 | 61 ± 41 | 0.32 ± 0.01 | 0.90 ± 0.01 |
| 3 mo | 26 ± 13 | 21 ± 15 | 0.33 ± 0.13 | 29 ± 33 | 0.30 ± 0.03 | 0.81 ± 0.04 |
| 4 mo | 17 ± 9 | 19 ± 2 | 0.27 ± 0.12 | 40 ± 3 | 0.27 ± 0.03 | 0.82 ± 0.12 |
|  | *MU Female* | | | | | |
| 1 mo | 14 ± 6 | 14 ± 12 | 0.65 ± 0.46 | 21 ± 22 | 0.31 ± 0.15 | 0.77 ± 0.07 |
| 2 mo | 23 ± 12 | 8 ± 9 | 0.98 ± 0.67 | 31 ± 36 | 0.22 ± 0.12 | 0.73 ± 0.11 |
| 3 mo | 16 ± 11 | 9 ± 6 | 0.50 ± 0.26 | 51 ± 38 | 0.19 ± 0.08 | 0.71 ± 0.08 |
| 4 mo | 14 ± 5 | 6 ± 4 | 0.92 ± 0.41 | 35 ± 18 | 0.22 ± 0.11 | 0.71 ± 0.06 |

**Table S1B**. Average value of fitted HGO material parameters for DSC (N = 4 – 10/group). The R^2^ shows the goodness of fit. Experimental data came from [9-10].

| **DSC** | *WT Male* | | | | | |
| --- | --- | --- | --- | --- | --- | --- |
|  | ***c (kPa)*** | ***k_1_ (kPa)*** | ***k_2_*** | ***α (ᵒ)*** | ***κ*** | ***R^2^*** |
| 1 mo | 39 ± 4 | 13 ± 3 | 3.63 ± 2.20 | 90 ± 0.02 | 0.25 ± 0.02 | 0.86 ± 0.02 |
| 2 mo | 9 ± 1 | 38 ± 13 | 0.28 ± 0.30 | 23 ± 39 | 0.27 ± 0.03 | 0.83 ± 0.03 |
| 3 mo | 38 ± 24 | 34 ± 19 | 1.98 ± 1.88 | 68 ± 34 | 0.30 ± 0.02 | 0.80 ± 0.05 |
| 4 mo | 31 ± 32 | 37 ± 32 | 0.80 ±0.71 | 32 ± 35 | 0.26 ± 0.09 | 0.74 ± 0.10 |
|  | *MU Male* | | | | | |
| 1 mo | 13 ± 12 | 20 ± 9 | 0.54 ± 0.31 | 38 ± 31 | 0.25 ± 0.03 | 0.66 ± 0.05 |
| 2 mo | 13 ± 7 | 18 ± 13 | 0.56 ± 2.61 | 22 ± 39 | 0.29 ± 0.02 | 0.67 ± 0.10 |
| 3 mo | 26 ± 11 | 9 ± 7 | 0.50 ± 3.39 | 75 ± 34 | 0.28 ± 0.04 | 0.69 ± 0.06 |
| 4 mo | 16 ± 5 | 30 ± 17 | 0.33 ± 0.48 | 66 ± 19 | 0.25 ± 0.06 | 0.77 ± 0.01 |
|  | *WT Female* | | | | | |
| 1 mo | 20 ± 14 | 13 ± 9 | 0.81 ± 2.81 | 55 ± 35 | 0.21 ± 0.02 | 0.72 ± 0.07 |
| 2 mo | 19 ± 6 | 27 ± 14 | 0.62 ± 0.43 | 75 ± 25 | 0.29 ± 0.04 | 0.79 ± 0.05 |
| 3 mo | 23 ± 13 | 36 ± 32 | 0.23 ± 0.92 | 90 ± 1 | 0.30 ± 0.02 | 0.78 ± 0.05 |
| 4 mo | 16 ± 9 | 14 ± 6 | 0.83 ± 0.49 | 15 ± 22 | 0.31± 0.02 | 0.67 ± 0.21 |
|  | *MU Female* | | | | | |
| 1 mo | 21 ± 10 | 25 ± 30 | 0.66 ± 3.02 | 77 ± 21 | 0.27 ± 0.07 | 0.74 ± 0.06 |
| 2 mo | 16 ± 7 | 23 ± 18 | 0.72 ± 0.83 | 77 ± 29 | 0.31 ± 0.02 | 0.73 ± 0.07 |
| 3 mo | 28 ± 12 | 11 ± 10 | 0.48 ± 2.63 | 90 ± 1 | 0.27 ± 0.05 | 0.75 ± 0.08 |
| 4 mo | 23 ± 10 | 24 ± 4 | 0.69 ± 1.40 | 67 ± 17 | 0.28 ± 0.06 | 0.73 ± 0.07 |

**Table S1C**. Average values of HGO fitted material parameters for SAB (N = 2 – 8/group). The R^2^ shows the goodness of fit. Experimental data from Experimental data came from [9-10].

| **SAB** | *WT Male* | | | | | |
| --- | --- | --- | --- | --- | --- | --- |
|  | ***c (kPa)*** | ***k_1_ (kPa)*** | ***k_2_*** | ***α (ᵒ)*** | ***κ*** | ***R^2^*** |
| 1 mo | 24 ± 11 | 24 ± 10 | 1.03 ± 0.73 | 90 ± 1 | 0.26 ± 0.01 | 0.80 ± 0.01 |
| 2 mo | 12 ± 4 | 53 ± 11 | 0.94 ± 0.19 | 30 ± 42 | 0.26 ± 0.05 | 0.75 ± 0.10 |
| 3 mo | 15 ± 9 | 29 ± 14 | 0.72 ± 0.43 | 67 ± 39 | 0.28 ± 0.02 | 0.80 ± 0.03 |
| 4 mo | 38 ± 4 | 43 ± 9 | 1.43 ± 0.25 | 90 ± 1 | 0.19 ± 0.07 | 0.77 ± 0.04 |
|  | *MU Male* | | | | | |
| 1 mo | 29 ± 7 | 6 ± 3 | 1.28 ± 0.88 | 74 ± 16 | 0.20 ± 0.05 | 0.83 ± 0.05 |
| 2 mo | 13 ± 8 | 48 ± 42 | 1.22 ± 0.79 | 46 ± 16 | 0.27 ± 0.04 | 0.71 ± 0.04 |
| 3 mo | 9 ± 3 | 12 ± 7 | 2.70 ± 1.10 | 66 ± 33 | 0.26 ± 0.06 | 0.68 ± 0.12 |
| 4 mo | 5 ± 4 | 26 ± 20 | 3.15 ± 3.05 | 66 ± 18 | 0.24 ± 0.07 | 0.64 ± 0.10 |
|  | *WT Female* | | | | | |
| 1 mo | 24 ± 9 | 18 ± 2 | 1.70 ± 2.39 | 56 ± 4 | 0.23 ± 0.09 | 0.74 ± 0.03 |
| 2 mo | 21 ± 8 | 36 ± 8 | 0.32 ± 0.15 | 20 ± 23 | 0.28 ± 0.04 | 0.85 ± 0.03 |
| 3 mo | 38 ± 4 | 56 ± 44 | 1.40 ± 0.72 | 79 ± 11 | 0.13 ± 0.12 | 0.71 ± 0.12 |
| 4 mo | 13 ± 1 | 40 ± 4 | 0.32 ± 0.03 | 49 ± 7 | 0.28 ± 0.05 | 0.71 ± 0.01 |
|  | *MU Female* | | | | | |
| 1 mo | 22 ± 3 | 13 ± 4 | 1.06 ± 0.37 | 57 ± 24 | 0.24 ± 0.03 | 0.74 ± 0.06 |
| 2 mo | 28 ± 10 | 18 ± 21 | 3.07 ± 1.74 | 79 ± 30 | 0.24 ± 0.06 | 0.71 ± 0.08 |
| 3 mo | 16 ± 7 | 15 ± 7 | 1.98 ± 0.89 | 72 ± 36 | 0.30 ± 0.02 | 0.78 ± 0.02 |
| 4 mo | 28 ± 13 | 34 ± 13 | 1.13 ± 0.30 | 65 ± 25 | 0.25 ± 0.04 | 0.68 ± 0.13 |

**Table S1D**. Average values of HGO fitted material parameters for IAB (N = 2 – 8/group). The R^2^ shows the goodness of fit. Experimental data from Experimental data came from [9-10].

| **IAB** | *WT Male* | | | | | |
| --- | --- | --- | --- | --- | --- | --- |
|  | ***c (kPa)*** | ***k_1_ (kPa)*** | ***k_2_*** | ***α (ᵒ)*** | ***κ*** | ***R^2^*** |
| 1 mo | 32 ± 10 | 9 ± 9 | 3.96 ± 1.09 | 66 ± 23 | 0.18 ± 0.11 | 0.70 ± 0.07 |
| 2 mo | 14 ± 9 | 21 ± 15 | 1.06 ± 0.39 | 23 ± 39 | 0.27 ± 0.03 | 0.68 ± 0.09 |
| 3 mo | 21 ± 10 | 22 ± 13 | 3.09 ± 4.00 | 63 ± 36 | 0.24 ± 0.08 | 0.71 ± 0.07 |
| 4 mo | 16 ± 11 | 23 ± 23 | 0.47 ± 0.12 | 38 ± 43 | 0.17 ± 0.07 | 0.87 ± 0.03 |
|  | *MU Male* | | | | | |
| 1 mo | 23 ± 12 | 17 ± 6 | 0.51 ± 0.36 | 12 ± 19 | 0.25 ± 0.09 | 0.84 ± 0.09 |
| 2 mo | 21 ± 8 | 36 ± 8 | 0.32 ± 0.15 | 20 ± 23 | 0.28 ± 0.04 | 0.85 ± 0.03 |
| 3 mo | 32 ± 17 | 20 ± 13 | 0.24 ± 0.14 | 32 ± 40 | 0.30 ± 0.02 | 0.80 ± 0.08 |
| 4 mo | 30 ± 6 | 14 ± 6 | 0.36 ± 0.26 | 9 ± 17 | 0.30 ± 0.02 | 0.87 ± 0.03 |
|  | *WT Female* | | | | | |
| 1 mo | 21 ± 5 | 15 ± 3 | 0.55 ± 0.16 | 39. ± 1 | 0.23 ± 0.01 | 0.64 ± 0.04 |
| 2 mo | 14 ± 7 | 13 ± 9 | 2.06 ± 0.15 | 34 ± 37 | 0.28 ± 0.04 | 0.85 ± 0.03 |
| 3 mo | 15 ± 7 | 18 ± 8 | 1.29 ± 0.71 | 69 ± 21 | 0.29 ± 0.01 | 0.62 ± 0.03 |
| 4 mo | 18 ± 16 | 23 ± 9 | 1.95 ± 1.80 | 90 ± 1 | 0.22 ± 0.08 | 0.66 ± 0.02 |
|  | *MU Female* | | | | | |
| 1 mo | 17 ± 5 | 15 ± 3 | 0.86 ± 0.05 | 39 ± 31 | 0.23 ± 0.06 | 0.73 ± 0.01 |
| 2 mo | 25 ± 15 | 31 ± 30 | 2.18 ± 1.36 | 44 ± 40 | 0.25 ± 0.08 | 0.65 ± 0.08 |
| 3 mo | 15 ± 9 | 19 ± 12 | 2.28 ± 1.91 | 81 ± 18 | 0.27 ± 0.05 | 0.67 ± 0.09 |
| 4 mo | 50 ± 30 | 8 ± 7 | 1.80 ± 1.33 | 45 ± 45 | 0.28 ± 0.04 | 0.65 ± 0.01 |

**Table S2**. Average incremental modulus (*E_inc_*) corresponding to diastolic pressure for ASC, DSC, SAB and IAB used as the elasticity modulus for mesh stiffness initialization in the FSI simulation (N = 2 – 10/group). Experimental data from [9-10].

|  | *WT Male* | | | | *MU Male* | | | |
| --- | --- | --- | --- | --- | --- | --- | --- | --- |
|  | ASC | DSC | SAB | IAB | ASC | DSC | SAB | IAB |
|  | ***E_inc_ (kPa)*** | ***E_inc_ (kPa)*** | ***E_inc_ (kPa)*** | ***E_inc_ (kPa)*** | ***E_inc_ (kPa)*** | ***E_inc_ (kPa)*** | ***E_inc_ (kPa)*** | ***E_inc_ (kPa)*** |
| 1 mo | 597 | 569 | 603 | 992 | 1773 | 881 | 957 | 1328 |
| 2 mo | 576 | 509 | 590 | 935 | 1694 | 1206 | 1293 | 1126 |
| 3 mo | 613 | 487 | 576 | 1042 | 3413 | 1984 | 1589 | 1436 |
| 4 mo | 592 | 497 | 601 | 1477 | 6062 | 2086 | 1865 | 1845 |
|  | *WT Female* | | | | *MU Female* | | | |
| 1 mo | 503 | 621 | 575 | 1343 | 1620 | 912 | 738 | 1192 |
| 2 mo | 498 | 410 | 427 | 718 | 2216 | 1347 | 1307 | 1377 |
| 3 mo | 542 | 612 | 659 | 714 | 2128 | 1317 | 1339 | 1476 |
| 4 mo | 574 | 611 | 495 | 1159 | 2239 | 841 | 1176 | 1552 |

**Table S3A.** Mouse-specific MR-measured values of heart rate (HR), time-integrated velocity (VTI), and cross-sectional area at the aortic root for WT and MU male mice at different ages to determine inlet flow values (Fig. 1D).

|  |  | **WT Male** | | | |
| --- | --- | --- | --- | --- | --- |
| **Mouse ID** | **Age** | **1 mo** | **2 mo** | **3 mo** | **4 mo** |
| **37143_1M** | *HR (beats/sec)* | 395 | 405 | 387 | 364 |
|  | *VTI (mm/sec)* | 30.17 | 29.92 | 29.00 | 36.11 |
|  | *Flow area (mm2)* | 1.06 | 1.07 | 1.12 | 1.15 |
| **37145_2M** | *HR (beats/sec)* | 404 | 414 | 395 | 372 |
|  | *VTI (mm/sec)* | 31.07 | 30.81 | 29.86 | 37.19 |
|  | *Flow area (mm2)* | 0.85 | 1.04 | 1.11 | 1.15 |
| **37191_1M** | *HR (beats/sec)* | 393 | 403 | 385 | 362 |
|  | *VTI (mm/sec)* | 30.00 | 29.62 | 28.71 | 35.75 |
|  | *Flow area (mm2)* | 1.20 | `1.09 | 1.12 | 1.13 |
|  |  | **MU Male** | | | |
| **Mouse ID** | **Age** | **1 mo** | **2 mo** | **3 mo** | **4 mo** |
| **37145_1M** | *HR (beats/sec)* | 411 | 417 | No data, mouse died b/t 2-3 mo | |
|  | *VTI (mm/sec)* | 27.37 | 29.99 |  |  |
|  | *Flow area (mm^2^)* | 1.93 | 6.79 |  |  |
| **37145_3M** | *HR (beats/sec)* | 395 | No data, mouse died b/t 1-2 mo | | |
|  | *VTI (mm/sec)* | 30.17 |  |  |  |
|  | *Flow area (mm^2^)* | 0.89 |  |  |  |
| **37189_1M** | *HR (beats/sec)* | 415 | 408 | 397 | 415 |
|  | *VTI (mm/sec)* | 26.17 | 31.29 | 26.12 | 30.23 |
|  | *Flow area (mm^2^)* | 1.17 | 1.23 | 1.25 | 1.35 |
| **37226_2M** | *HR (beats/sec)* | 388 | 435 | 408 | 435 |
|  | *VTI (mm/sec)* | 31.17 | 27.92 | 24.11 | 28.21 |
|  | *Flow area (mm^2^)* | 0.83 | 1.00 | 1.33 | 1.91 |
| **37227_1M** | *HR (beats/sec)* | 442 | 437 | 410 | 425 |
|  | *VTI (mm/sec)* | 24.37 | 27.99 | 26.11 | 29.42 |
|  | *Flow area (mm^2^)* | 0.93 | 1.13 | 2.24 | 2.83 |
| **37227_2M** | *HR (beats/sec)* | 431 | 427 | No data, mouse died b/t 2-3 mo | |
|  | *VTI (mm/sec)* | 24.37 | 28.99 |  |  |
|  | *Flow area (mm2)* | 1.58 | 2.53 |  |  |

**Table S3B.** Mouse-specific MR-measured values of heart rate (HR), time-integrated velocity (VTI), and cross-sectional area at the aortic root for WT and MU female mice at different ages to determine inlet flow values (Fig. 1D).

|  |  | **WT_Female** | | | |
| --- | --- | --- | --- | --- | --- |
| **Mouse ID** | **Age** | **1 mo** | **2 mo** | **3 mo** | **4 mo** |
| **37144_1F** | *HR (beats/sec)* | 390 | 438 | 426 | 441 |
|  | *VTI (mm/sec)* | 31.00 | 26.00 | 32.00 | 29.00 |
|  | *Flow area (mm^2^)* | 0.65 | 0.74 | 1.04 | 1.06 |
| **37144_3F** | *HR (beats/sec)* | 398 | 430 | 410 | 365 |
|  | *VTI (mm/sec)* | 30.50 | 27.00 | 28.00 | 31.00 |
|  | *Flow area (mm^2^)* | 0.65 | 1.02 | 1.13 | 1.09 |
| **37190_2F** | *HR (beats/sec)* | 398 | 431 | 402 | 388 |
|  | *VTI (mm/sec)* | 30.00 | 27.00 | 32.00 | 29.00 |
|  | *Flow area (mm^2^)* | 0.87 | 0.97 | 1.07 | 1.09 |
|  |  | **MU_Female** | | | |
| **Mouse ID** | **Age** | **1 mo** | **2 mo** | **3 mo** | **4 mo** |
| **37188_1F** | *HR (beats/sec)* | 398 | 404 | 412 | 435 |
|  | *VTI (mm/sec)* | 25.00 | 29.57 | 31.95 | 24.64 |
|  | *Flow area (mm^2^)* | 1.04 | 1.12 | 1.19 | 1.23 |
| **37190_1F** | *HR (beats/sec)* | 408 | 414 | 438 | 425 |
|  | *VTI (mm/sec)* | 24.51 | 28.64 | 30.00 | 25.15 |
|  | *Flow area (mm^2^)* | 0.97 | 1.00 | 1.06 | 1.12 |
| **37192_1F** | *HR (beats/sec)* | 411 | 420 | 428 | 422 |
|  | *VTI (mm/sec)* | 24.35 | 26.21 | 31.00 | 24.48 |
|  | *Flow area (mm^2^)* | 1.02 | 1.13 | 1.13 | 1.12 |
| **37192_1F** | *HR (beats/sec)* | 390 | 424 | 416 | No data, mouse died b/t 3-4 months of age |
|  | *VTI (mm/sec)* | 25.88 | 28.14 | 31.00 |  |
|  | *Flow area (mm^2^)* | 1.06 | 1.09 | 1.13 |  |
| **37146_1F** | *HR (beats/sec)* | 402 | 414 | 432 | No data, mouse died b/t 3-4 months of age |
|  | *VTI (mm/sec)* | 24.91 | 28.54 | 31.00 |  |
|  | *Flow area (mm^2^)* | 1.04 | 1.39 | 1.58 |  |

**Table S4.** Systolic and diastolic pressures for each age, sex and genotype (mean ± SD) from talk cuff measurements (Fig. 1I). Data from [9].

|  | **WT Male** | | **MU Male** | |
| --- | --- | --- | --- | --- |
|  | **Sys Pressure (mmHg)** | **Dias Pressure (mmHg)** | **Sys Pressure (mmHg)** | **Dias Pressure (mmHg)** |
| 1 mo | 113 ± 8 | 86 ± 6 | 118 ± 11 | 89 ± 10 |
| 2 mo | 121 ± 5 | 92 ± 7 | 122 ± 9 | 86 ± 10 |
| 3 mo | 128 ± 11 | 92 ± 14 | 12 ± 13 | 89 ± 5 |
| 4 mo | 127 ± 6 | 96 ± 7 | 127 ± 3 | 88 ± 5 |
|  | **WT Female** | | **MU Female** | |
|  | **Sys Pressure (mmHg)** | **Dias Pressure (mmHg)** | **Sys Pressure (mmHg)** | **Dias Pressure (mmHg)** |
| 1 mo | 116 ± 5 | 87 ± 5 | 114 ± 8 | 86 ± 4 |
| 2 mo | 132 ± 12 | 94 ± 9 | 130 ± 15 | 94 ± 14 |
| 3 mo | 126 ± 18 | 96 ± 21 | 123 ± 18 | 88 ± 18 |
| 4 mo | 126 ± 9 | 89 ± 13 | 124 ± 16 | 91 ± 18 |

**Table S5A**. *R-C-r* (Fig. 1E) values each aortic outlet (Fig. 1C) for each WT male mouse.

|  |  | **WT Male** | | | | | | | | | | | | | |
| --- | --- | --- | --- | --- | --- | --- | --- | --- | --- | --- | --- | --- | --- | --- | --- |
|  | **Age** | **1 mo** | | | **2 mo** | | | **3 mo** | | | | **4 mo** | | | |
| **Mouse ID** | **Outlet** | **R (Pa-s/mm^3^)** | **C (mm^3^/Pa)** | **r (Pa-s/mm^3^)** | **R (Pa-s/mm^3^)** | **C (mm^3^/Pa)** | **r (Pa-s/mm^3^)** | **R (Pa-s/mm^3^)** | **C (mm^3^/Pa)** | **r (Pa-s/mm^3^)** | **R (Pa-s/mm^3^)** | | **C (mm^3^/Pa)** | **r (Pa-s/mm^3^)** |  |
| **39143_1M** | 1 | 15.58 | 1.21E-03 | 113.17 | 15.58 | 1.21E-03 | 113.17 | 2.37 | 4.06E-04 | 142.68 | 0.94 | | 8.09E-04 | 92.30 |  |
|  | 2 | 12.08 | 1.86E-04 | 585.05 | 12.08 | 1.86E-04 | 585.05 | 2.69 | 1.22E-03 | 590.69 | 1.80 | | 6.01E-04 | 388.86 |  |
|  | 3 | 39.64 | 1.46E-03 | 810.89 | 39.64 | 1.46E-03 | 810.89 | 2.76 | 6.09E-04 | 656.75 | 1.39 | | 2.67E-04 | 435.40 |  |
|  | 4 | 107.14 | 1.10E-03 | 845.82 | 107.14 | 1.10E-03 | 845.82 | 8.21 | 1.07E-03 | 600.48 | 3.12 | | 5.09E-04 | 427.22 |  |
|  | 5 | 20.21 | 1.28E-03 | 1648.95 | 20.21 | 1.28E-03 | 1648.96 | 11.57 | 1.02E-03 | 496.97 | 3.21 | | 1.03E-03 | 447.02 |  |
| **39145_2M** | 1 | 12.49 | 2.58E-03 | 230.88 | 11.38 | 1.13E-03 | 173.40 | 1.57 | 2.48E-03 | 118.84 | 4.14 | | 1.43E-03 | 120.42 |  |
|  | 2 | 5.60 | 1.31E-03 | 862.69 | 7.86 | 1.54E-03 | 667.71 | 1.77 | 1.37E-03 | 536.80 | 7.09 | | 2.48E-04 | 679.77 |  |
|  | 3 | 17.95 | 1.93E-03 | 1535.12 | 21.33 | 9.24E-04 | 1463.46 | 10.49 | 2.65E-03 | 710.83 | 7.63 | | 1.40E-03 | 811.66 |  |
|  | 4 | 12.43 | 2.36E-03 | 1193.90 | 10.19 | 2.24E-03 | 979.22 | 19.78 | 1.22E-03 | 459.55 | 15.75 | | 1.18E-03 | 682.25 |  |
|  | 5 | 19.01 | 7.42E-04 | 737.05 | 18.62 | 1.94E-03 | 729.22 | 6.12 | 9.53E-04 | 597.54 | 19.35 | | 1.12E-03 | 520.59 |  |
| **39191_1M** | 1 | 12.81 | 1.05E-03 | 124.63 | 5.96 | 1.28E-03 | 112.58 | 8.78 | 6.77E-04 | 105.40 | 1.17 | | 6.53E-04 | 94.58 |  |
|  | 2 | 12.39 | 1.24E-03 | 610.87 | 6.52 | 1.20E-03 | 358.96 | 3.23 | 3.78E-04 | 481.87 | 4.22 | | 7.50E-04 | 375.07 |  |
|  | 3 | 11.56 | 1.44E-03 | 806.14 | 5.46 | 3.83E-04 | 504.86 | 10.44 | 6.59E-04 | 671.34 | 7.03 | | 1.12E-03 | 532.45 |  |
|  | 4 | 1.74 | 2.39E-04 | 664.05 | 1.01 | 6.11E-04 | 380.72 | 9.97 | 7.50E-04 | 585.23 | 7.60 | | 1.10E-03 | 353.32 |  |
|  | 5 | 5.25 | 2.81E-04 | 548.70 | 1.79 | 4.57E-04 | 304.51 | 12.85 | 1.36E-03 | 536.33 | 1.16 | | 9.06E-04 | 364.14 |  |

**Table S5B**. *R-C-r* (Fig. 1E) values each aortic outlet (Fig. 1C) for each WT female mouse.

|  |  | **WT Female** | | | | | | | | | | | |
| --- | --- | --- | --- | --- | --- | --- | --- | --- | --- | --- | --- | --- | --- |
|  | **Age** | **1 mo** | | | **2 mo** | | | **3 mo** | | | **4 mo** | | |
| **Mouse ID** | **Outlet** | **R (Pa-s/mm^3^)** | **C (mm^3^/Pa)** | **r (Pa-s/mm^3^)** | **R (Pa-s/mm^3^)** | **C (mm^3^/Pa)** | **r (Pa-s/mm^3^)** | **R (Pa-s/mm^3^)** | **C (mm^3^/Pa)** | **r (Pa-s/mm^3^)** | **R (Pa-s/mm^3^)** | **C (mm^3^/Pa)** | **r (Pa-s/mm^3^)** |
| **37144_1F** | 1 | 12.49 | 2.58E-03 | 230.88 | 11.38 | 1.13E-03 | 173.40 | 1.57 | 2.48E-03 | 118.84 | 4.14 | 1.43E-03 | 120.42 |
|  | 2 | 5.60 | 1.32E-03 | 862.69 | 7.86 | 1.54E-03 | 667.71 | 1.77 | 1.37E-03 | 536.80 | 7.09 | 2.48E-04 | 679.77 |
|  | 3 | 17.95 | 1.93E-03 | 1535.12 | 21.33 | 9.24E-04 | 1463.46 | 10.49 | 2.65E-03 | 710.83 | 7.63 | 1.40E-03 | 811.66 |
|  | 4 | 12.43 | 2.36E-03 | 1193.90 | 10.19 | 2.24E-03 | 979.22 | 19.78 | 1.22E-03 | 459.55 | 15.75 | 1.18E-03 | 682.25 |
|  | 5 | 19.01 | 7.42E-04 | 737.05 | 18.62 | 1.94E-03 | 729.22 | 6.12 | 9.53E-04 | 597.54 | 19.35 | 1.12E-03 | 520.59 |
| **37144_3F** | 1 | 6.87 | 2.56E-03 | 202.66 | 3.02 | 2.23E-03 | 144.44 | 3.05 | 0.002252 | 145.89 | 8.32 | 1.24E-03 | 120.59 |
|  | 2 | 3.44 | 1.76E-03 | 915.54 | 4.46 | 1.40E-03 | 476.86 | 4.50 | 0.001414 | 481.63 | 11.39 | 1.18E-03 | 658.12 |
|  | 3 | 3.80 | 1.57E-03 | 1195.57 | 2.98 | 1.05E-03 | 861.67 | 3.01 | 0.001061 | 870.29 | 5.43 | 1.01E-03 | 762.29 |
|  | 4 | 17.69 | 2.36E-03 | 1023.94 | 11.86 | 1.73E-03 | 588.16 | 11.98 | 0.001747 | 594.04 | 5.13 | 5.15E-04 | 615.78 |
|  | 5 | 26.28 | 1.85E-03 | 781.41 | 21.24 | 1.52E-03 | 529.22 | 21.46 | 0.001535 | 534.51 | 26.82 | 4.39E-04 | 457.11 |
| **37190_2F** | 1 | 8.39 | 1.47E-03 | 151.88 | 3.11 | 7.59E-04 | 117.92 | 8.26 | 1.59E-03 | 107.10 | 8.72 | 2.51E-03 | 141.20 |
|  | 2 | 8.19 | 2.21E-03 | 652.39 | 2.62 | 1.55E-03 | 679.40 | 1.43 | 6.27E-04 | 520.93 | 3.40 | 2.05E-03 | 412.84 |
|  | 3 | 15.44 | 3.98E-04 | 1223.22 | 4.40 | 9.14E-04 | 788.78 | 3.05 | 1.31E-03 | 519.63 | 20.72 | 1.52E-03 | 598.00 |
|  | 4 | 9.90 | 1.93E-03 | 848.66 | 12.73 | 4.45E-04 | 650.05 | 4.20 | 3.36E-04 | 509.31 | 18.70 | 7.38E-04 | 665.84 |
|  | 5 | 5.83 | 8.14E-04 | 487.66 | 5.59 | 8.50E-04 | 528.23 | 17.02 | 1.94E-03 | 421.11 | 23.29 | 2.35E-03 | 552.88 |

|  |  | **MU Male** | | | | | | | | | | | |
| --- | --- | --- | --- | --- | --- | --- | --- | --- | --- | --- | --- | --- | --- |
|  | **Age** | **1 mo** | | | **2 mo** | | | **3 mo** | | | **4 mo** | | |
| **Mouse ID** | **Outlet** | **R (Pa-s/mm^3^)** | **C (mm^3^/Pa)** | **r (Pa-s/mm^3^)** | **R (Pa-s/mm^3^)** | **C (mm^3^/Pa)** | **r (Pa-s/mm^3^)** | **R (Pa-s/mm^3^)** | **C (mm^3^/Pa)** | **r (Pa-s/mm^3^)** | **R (Pa-s/mm^3^)** | **C (mm^3^/Pa)** | **r (Pa-s/mm^3^)** |
| **37189_1M** | 1 | 7.18 | 1.37E-03 | 112.95 | 1.92 | 0.001059 | 97.96 | 7.97 | 5.47E-04 | 110.87 | 3.34 | 1.76E-03 | 83.68 |
|  | 2 | 6.07 | 1.14E-03 | 472.01 | 0.78 | 0.001106 | 432.05 | 13.61 | 1.24E-03 | 463.19 | 1.99 | 1.81E-03 | 335.63 |
|  | 3 | 1.42 | 8.24E-04 | 612.65 | 0.57 | 0.000667 | 632.37 | 4.86 | 2.26E-04 | 748.17 | 1.24 | 4.83E-04 | 404.66 |
|  | 4 | 1.34 | 4.40E-04 | 663.12 | 0.56 | 0.000983 | 490.55 | 2.52 | 1.21E-03 | 682.70 | 14.72 | 2.14E-03 | 374.69 |
|  | 5 | 2.45 | 5.59E-04 | 596.10 | 2.66 | 0.001075 | 360.29 | 1.62 | 3.96E-04 | 484.61 | 9.23 | 5.33E-04 | 268.64 |
| **37145_1M** | 1 | 8.06 | 1.37E-03 | 62.41 | 1.12 | 1.08E-02 | 28.39 | No data, mouse died b/t 2-3 mo | | | | | |
|  | 2 | 5.16 | 8.88E-04 | 280.09 | 0.98 | 6.61E-03 | 76.70 |  |  |  |  |  |  |
|  | 3 | 6.29 | 1.26E-03 | 352.86 | 0.11 | 2.60E-03 | 207.24 |  |  |  |  |  |  |
|  | 4 | 5.32 | 1.11E-03 | 317.91 | 0.77 | 4.74E-03 | 83.23 |  |  |  |  |  |  |
|  | 5 | 5.09 | 1.38E-03 | 202.31 | 0.61 | 5.26E-03 | 61.54 |  |  |  |  |  |  |
| **37145_3M** | 1 | 4.65 | 1.35E-03 | 77.31 | No data, mouse died b/t 1-2 mo | | | | | | | | |
|  | 2 | 2.12 | 5.95E-04 | 382.33 |  |  |  |  |  |  |  |  |  |
|  | 3 | 4.69 | 3.95E-04 | 498.99 |  |  |  |  |  |  |  |  |  |
|  | 4 | 4.33 | 5.74E-04 | 391.89 |  |  |  |  |  |  |  |  |  |
|  | 5 | 6.60 | 5.12E-04 | 282.35 |  |  |  |  |  |  |  |  |  |
| **37226_2M** | 1 | 3.82 | 6.57E-04 | 162.42 | 3.50 | 9.36E-04 | 116.06 | 8.54 | 5.23E-04 | 89.30 | 4.49 | 1.45E-03 | 61.46 |
|  | 2 | 2.67 | 2.08E-04 | 766.44 | 1.52 | 8.80E-04 | 332.81 | 2.42 | 1.15E-03 | 445.43 | 7.71 | 1.08E-03 | 244.52 |
|  | 3 | 7.18 | 1.47E-03 | 784.85 | 3.60 | 1.35E-03 | 531.16 | 6.11 | 3.47E-04 | 540.14 | 4.04 | 1.05E-03 | 298.63 |
|  | 4 | 9.22 | 7.03E-04 | 871.97 | 0.91 | 8.56E-04 | 391.74 | 1.12 | 1.26E-03 | 386.67 | 7.76 | 9.37E-04 | 302.55 |
|  | 5 | 8.24 | 9.89E-04 | 590.09 | 2.33 | 1.34E-03 | 513.26 | 5.59 | 1.16E-03 | 484.21 | 3.62 | 3.34E-04 | 237.96 |
| **37227_1M** | 1 | 6.63 | 1.39E-03 | 139.14 | 9.49 | 1.30E-03 | 96.13 | 4.07 | 1.25E-03 | 61.72 | 0.70 | 1.85E-03 | 50.91 |
|  | 2 | 7.46 | 5.17E-04 | 610.92 | 1.43 | 1.10E-03 | 450.91 | 1.14 | 8.77E-04 | 256.16 | 1.68 | 3.12E-04 | 187.54 |
|  | 3 | 6.66 | 8.79E-04 | 976.78 | 10.28 | 5.42E-04 | 486.20 | 3.25 | 8.20E-04 | 410.73 | 0.43 | 2.31E-03 | 385.11 |
|  | 4 | 9.92 | 6.01E-04 | 878.24 | 7.54 | 8.92E-04 | 563.52 | 1.05 | 5.40E-04 | 344.38 | 0.50 | 7.50E-04 | 190.82 |
|  | 5 | 9.81 | 3.06E-04 | 671.84 | 3.54 | 1.30E-03 | 458.96 | 1.69 | 1.20E-03 | 212.99 | 0.08 | 2.10E-03 | 144.84 |
| **37227_2M** | 1 | 0.81 | 1.42E-03 | 76.30 | 3.86 | 7.91E-04 | 88.46 | No data, mouse died b/t 2-3 mo | | | | | |
|  | 2 | 4.77 | 1.02E-03 | 396.37 | 2.73 | 7.13E-04 | 418.47 |  |  |  |  |  |  |
|  | 3 | 4.08 | 7.86E-04 | 458.81 | 3.43 | 3.83E-04 | 510.44 |  |  |  |  |  |  |
|  | 4 | 0.88 | 8.51E-04 | 381.81 | 4.43 | 7.76E-04 | 366.71 |  |  |  |  |  |  |
|  | 5 | 0.73 | 6.67E-04 | 338.52 | 4.37 | 1.13E-03 | 327.81 |  |  |  |  |  |  |

**Table S5C.** *R-C-r* (Fig. 1E) values each aortic outlet (Fig. 1C) for each MU male mouse.

**Table S5D.** *R-C-r* (Fig. 1E) values each aortic outlet (Fig. 1C) for each MU female mouse.

|  |  | **MU Male** | | | | | | | | | | | | | |
| --- | --- | --- | --- | --- | --- | --- | --- | --- | --- | --- | --- | --- | --- | --- | --- |
|  | **Age** | **1 mo** | | | **2 mo** | | | **3 mo** | | | | **4 mo** | | | |
| **Mouse ID** | **Outlet** | **R (Pa-s/mm3)** | **C (mm3/Pa)** | **r (Pa-s/mm3)** | **R (Pa-s/mm3)** | **C (mm3/Pa)** | **r (Pa-s/mm3)** | **R (Pa-s/mm3)** | **C (mm3/Pa)** | **r (Pa-s/mm3)** | **R (Pa-s/mm3)** | | **C (mm3/Pa)** | **r (Pa-s/mm3)** |  |
| **37188_1F** | 1 | 15.14 | 2.05E-03 | 111.23 | 11.62 | 1.31E-03 | 104.19 | 1.22 | 1.45E-03 | 102.36 | 5.67 | | 3.66E-04 | 107.53 |  |
|  | 2 | 9.59 | 1.26E-03 | 507.80 | 7.63 | 1.25E-03 | 373.05 | 6.38 | 1.36E-03 | 332.43 | 10.33 | | 6.72E-04 | 476.85 |  |
|  | 3 | 5.10 | 8.41E-04 | 683.29 | 11.81 | 1.09E-03 | 749.06 | 5.97 | 1.44E-03 | 527.69 | 6.54 | | 1.42E-03 | 711.65 |  |
|  | 4 | 8.16 | 2.33E-03 | 836.97 | 2.20 | 1.43E-04 | 573.88 | 8.25 | 9.83E-04 | 500.62 | 4.56 | | 2.87E-04 | 696.52 |  |
|  | 5 | 14.34 | 1.86E-03 | 558.49 | 11.93 | 5.23E-04 | 544.64 | 3.24 | 2.87E-04 | 307.36 | 6.45 | | 9.66E-04 | 443.78 |  |
| **37146_1F** | 1 | 10.76 | 1.44E-03 | 140.14 | 6.53 | 9.04E-04 | 74.09 | 8.09 | 8.91E-04 | 67.83 | No data, mouse died b/t 3-4 mo | | | |  |
|  | 2 | 2.90 | 1.94E-04 | 591.38 | 3.11 | 7.45E-04 | 368.25 | 4.65 | 7.99E-04 | 356.26 |  |  |  |  |  |
|  | 3 | 13.16 | 4.37E-04 | 826.51 | 1.18 | 1.19E-03 | 503.48 | 7.09 | 9.29E-04 | 518.69 |  |  |  |  |  |
|  | 4 | 4.96 | 1.05E-03 | 889.97 | 3.97 | 1.16E-03 | 445.57 | 7.52 | 1.17E-03 | 381.47 |  |  |  |  |  |
|  | 5 | 12.91 | 9.52E-04 | 552.13 | 8.85 | 1.36E-03 | 410.72 | 6.89 | 1.38E-03 | 355.44 |  |  |  |  |  |
| **37192_2F** | 1 | 11.80 | 2.02E-03 | 135.22 | 8.72 | 2.51E-03 | 141.20 | 5.59 | 3.61E-04 | 97.53 | No data, mouse died b/t 3-4 mo | | | |  |
|  | 2 | 10.27 | 1.32E-03 | 711.43 | 3.40 | 2.05E-03 | 412.84 | 0.93 | 4.99E-04 | 454.48 |  |  |  |  |  |
|  | 3 | 18.51 | 1.64E-03 | 1105.44 | 20.72 | 1.52E-03 | 598.00 | 3.18 | 1.45E-03 | 569.57 |  |  |  |  |  |
|  | 4 | 5.66 | 2.12E-03 | 741.59 | 18.70 | 7.38E-04 | 665.84 | 0.58 | 7.84E-04 | 659.87 |  |  |  |  |  |
|  | 5 | 31.76 | 1.05E-03 | 506.48 | 23.29 | 2.35E-03 | 552.88 | 1.60 | 1.25E-03 | 512.80 |  |  |  |  |  |
| **37192_1F** | 1 | 1.33 | 2.61E-03 | 102.47 | 4.30 | 1.64E-03 | 109.41 | 3.49 | 2.19E-03 | 99.83 | 3.91 | | 1.99E-03 | 109.81 |  |
|  | 2 | 13.70 | 2.15E-03 | 525.08 | 1.71 | 4.61E-04 | 511.38 | 5.05 | 2.55E-03 | 419.34 | 5.66 | | 2.32E-03 | 461.27 |  |
|  | 3 | 3.87 | 1.81E-03 | 732.90 | 5.41 | 6.75E-04 | 640.61 | 5.05 | 2.45E-03 | 559.02 | 5.65 | | 2.23E-03 | 614.92 |  |
|  | 4 | 20.34 | 6.32E-04 | 491.44 | 1.87 | 3.04E-04 | 695.35 | 5.35 | 1.71E-03 | 501.48 | 6.00 | | 1.55E-03 | 551.62 |  |
|  | 5 | 13.55 | 1.38E-03 | 418.71 | 15.98 | 2.54E-03 | 410.42 | 7.97 | 2.45E-03 | 468.45 | 8.92 | | 2.23E-03 | 515.30 |  |
| **37190_1F** | 1 | 1.33 | 2.61E-03 | 102.47 | 4.30 | 1.64E-03 | 109.41 | 3.49 | 2.19E-03 | 99.83 | 7.47 | | 1.57E-03 | 79.12 |  |
|  | 2 | 13.70 | 2.15E-03 | 525.08 | 1.71 | 4.61E-04 | 511.38 | 5.05 | 2.55E-03 | 419.34 | 2.03 | | 2.31E-03 | 397.37 |  |
|  | 3 | 3.87 | 1.81E-03 | 732.90 | 5.41 | 6.75E-04 | 640.61 | 5.05 | 2.45E-03 | 559.02 | 5.57 | | 2.14E-03 | 506.67 |  |
|  | 4 | 20.34 | 6.32E-04 | 491.44 | 1.87 | 3.04E-04 | 695.35 | 5.35 | 1.71E-03 | 501.48 | 8.08 | | 1.18E-03 | 488.36 |  |
|  | 5 | 13.55 | 1.38E-03 | 418.71 | 15.98 | 2.54E-03 | 410.42 | 7.97 | 2.45E-03 | 468.45 | 16.16 | | 1.50E-03 | 364.47 |  |

**Table S6A**, Pairwise comparison for maximum diameter of ASC between genotype or sex (contrast) (Fig. 2A). P value indicates the statistical significance difference.


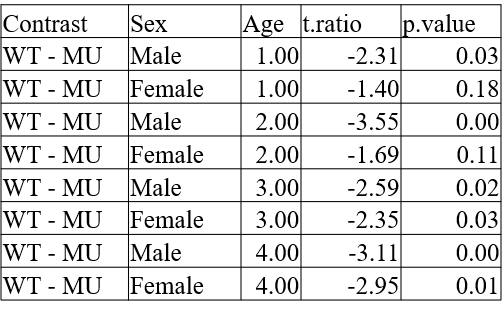

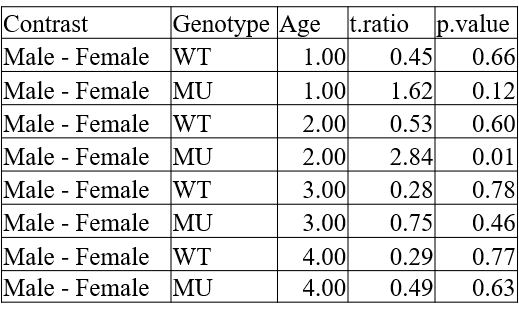


**Table S6B**. Pairwise comparison for AER between genotype or sex (contrast) (Fig. 2B). P value indicates the statistical significance difference.


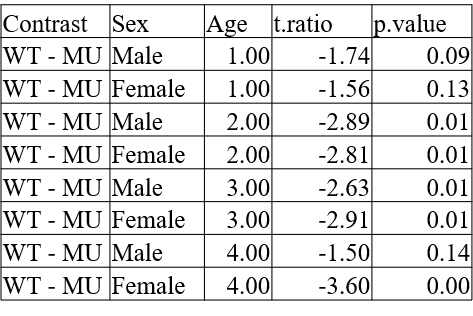

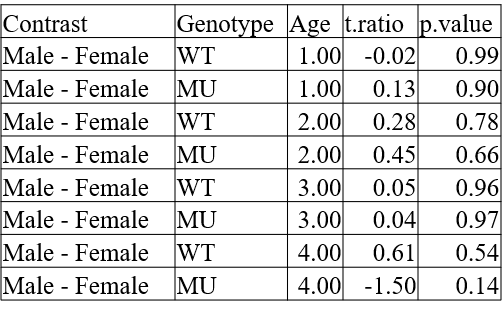


**Table S7**. Pairwise comparison for elastic fiber porosity between genotype or sex (contrast) (Fig. 3E). P value indicates the statistical significance difference.


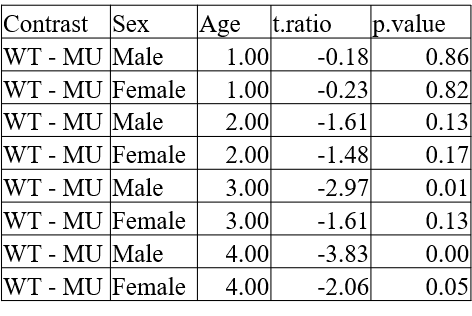

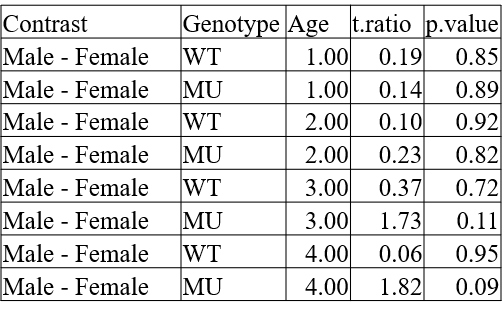


**Table S8A**. Pairwise comparison for mean OSI between genotype or sex (contrast) (Fig. 5A). P value indicates the statistical significance difference.


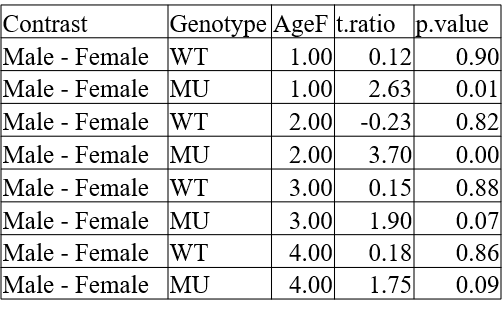

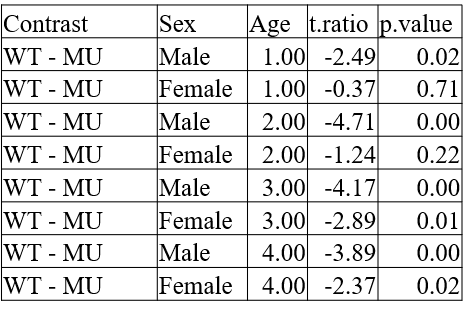


**Table S8B**. Pairwise comparison for mean TAWSS between genotype or sex (contrast) (Fig. 5B). P value indicates the statistical significance difference.


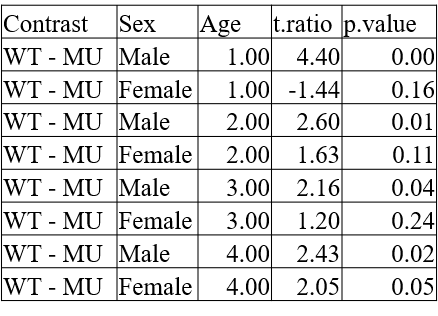

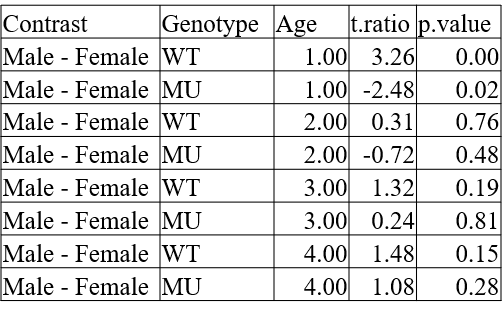


**Table S8C**. Pairwise comparison for mean ECAP between genotype or sex (contrast) (Fig. 5C). P value indicates the statistical significance difference.


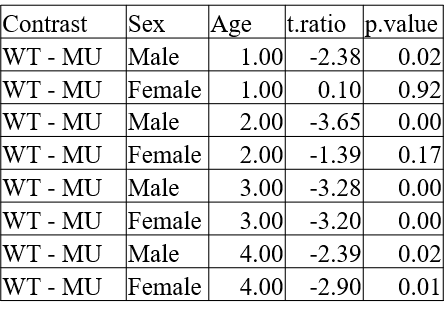

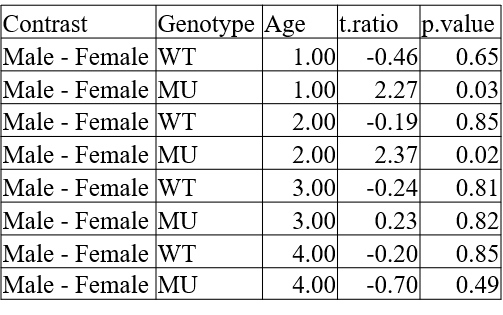


**Table S8D**. Pairwise comparison for mean RRT between genotype or sex (contrast) (Fig. 5D). P value indicates the statistical significance difference.


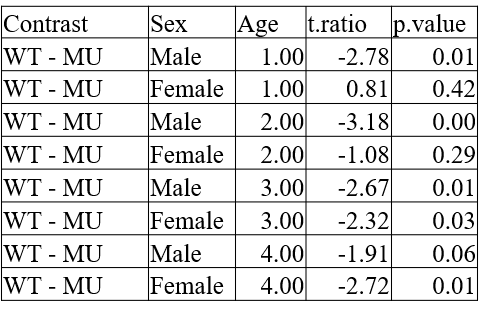

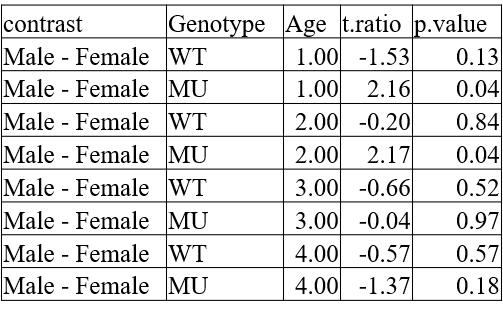


**Table S8E**. Pairwise comparison for mean systolic von Mises stress between genotype or sex (contrast) (Fig. 5E). P value indicates the statistical significance difference.


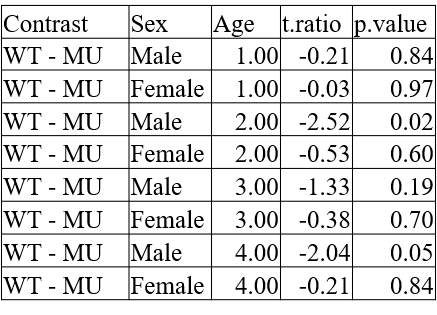

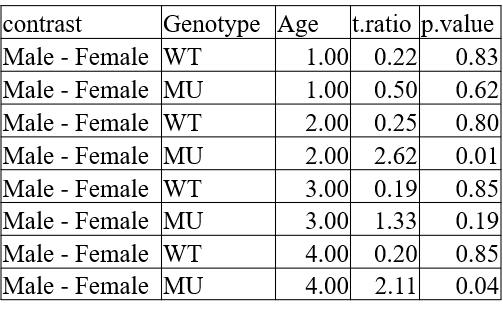


**Table S9** Spearman’s correlation results between OSI, TAWSS, ECAP and RRT. The r and p value indicates the correlation strength and statistical significance level.

|  | **WT Males** | | **MU Males** | | **WT Females** | | **MU Males** | |
| --- | --- | --- | --- | --- | --- | --- | --- | --- |
| **Parameters** | r-value | p-value | r-value | p-value | r-value | p-value | r-value | p-value |
| OSI vs TAWSS | -0.45 | 0.1404 | -0.39 | 0.118 | -0.42 | 0.1767 | -0.41 | 0.0925 |
| OSI vs ECAP | 0.73 | 0.01 | 0.91 | <0.0001 | 0.62 | 0.032 | 0.91 | <0.0001 |
| OSI vs RRT | 0.51 | 0.0936 | 0.81 | 0.0001 | 0.46 | 0.134 | -0.95 | <0.0001 |
| TAWSS vs ECAP | -0.86 | 0.0007 | -0.47 | 0.06 | -0.82 | 0.0019 | -0.83 | <0.0001 |
| TAWSS vs RRT | -0.99 | <0.0001 | -0.82 | <0.0001 | -0.95 | <0.0001 | -0.95 | <0.0001 |
| ECAP vs RRT | 0.89 | 0.0002 | 0.84 | <0.0001 | 0.83 | 0.0013 | 0.93 | <0.0001 |
